# BAP1 and YY1 regulate expression of death receptors in malignant pleural mesothelioma

**DOI:** 10.1016/j.jbc.2021.101223

**Published:** 2021-09-29

**Authors:** Yuki Ishii, Krishna K. Kolluri, Adam Pennycuick, Xidan Zhang, Ersilia Nigro, Doraid Alrifai, Elaine Borg, Mary Falzon, Khalid Shah, Neelam Kumar, Sam M. Janes

**Affiliations:** 1 Lungs for Living Research Centre, UCL Respiratory, University College London, London, United Kingdom; 2 Department of Environmental, Biological, and Pharmaceutical Sciences and Technologies, University of Campania Luigi Vanvitelli, Caserta, Italy; 3 Department of Histopathology, University College London Hospital, London, United Kingdom; 4 Center for Stem Cell Therapeutics and Imaging, Brigham and Women’s Hospital, Harvard Medical School, Boston, Massachusetts, USA

**Keywords:** BAP1, YY1, TRAIL, apoptosis, cancer therapy, tumor cell biology, receptor regulation, ASXL, additional sex combs-like, BAP1, BRCA1-associated protein 1, CCRCC, clear cell renal cell carcinoma, ChIP, chromatin immunoprecipitation, CK5, cytokeratin 5, co-IP, co-immunoprecipitation, DMEM, Dulbecco's modified Eagle's medium, DR4, death receptor 4, DR5, death receptor 5, DUB, deubiquitinase, FBS, fetal bovine serum, HBEC, human bronchial epithelial cell, HCF-1, host cell factor 1, IgG, immunoglobulin G, MPM, malignant pleural mesothelioma, PARP, poly(ADP-ribose) polymerase, qPCR, quantitative PCR, rTRAIL, recombinant tumor necrosis factor–related apoptosis-inducing ligand, TMA, tissue microarray, TNF, tumor necrosis factor, TRAIL, tumor necrosis factor–related apoptosis-inducing ligand, YY1, Ying Yang 1

## Abstract

Malignant pleural mesothelioma (MPM) is a rare, aggressive, and incurable cancer arising from the mesothelial lining of the pleura, with few available treatment options. We recently reported that loss of function of the nuclear deubiquitinase BRCA1-associated protein 1 (BAP1), a frequent event in MPM, is associated with sensitivity to tumor necrosis factor–related apoptosis-inducing ligand (TRAIL)–mediated apoptosis. As a potential underlying mechanism, here we report that BAP1 negatively regulates the expression of TRAIL receptors: death receptor 4 (DR4) and death receptor 5 (DR5). Using tissue microarrays of tumor samples from MPM patients, we found a strong inverse correlation between BAP1 and TRAIL receptor expression. BAP1 knockdown increased DR4 and DR5 expression, whereas overexpression of BAP1 had the opposite effect. Reporter assays confirmed wt-BAP1, but not catalytically inactive BAP1 mutant, reduced promoter activities of *DR4* and *DR5*, suggesting deubiquitinase activity is required for the regulation of gene expression. Co-immunoprecipitation studies demonstrated direct binding of BAP1 to the transcription factor Ying Yang 1 (YY1), and chromatin immunoprecipitation assays revealed BAP1 and YY1 to be enriched in the promoter regions of *DR4* and *DR5*. Knockdown of *YY1* also increased DR4 and DR5 expression and sensitivity to TRAIL. These results suggest that BAP1 and YY1 cooperatively repress transcription of TRAIL receptors. Our finding that BAP1 directly regulates the extrinsic apoptotic pathway will provide new insights into the role of BAP1 in the development of MPM and other cancers with frequent BAP1 mutations.

Malignant pleural mesothelioma (MPM) is a rare and aggressive cancer that arises from the mesothelial lining of the lungs and is commonly associated with occupational exposure to asbestos. There are currently no curative therapies. Standard first-line treatment is combination chemotherapy consisting of an antifolate and a platinum agent that offers only a modest survival benefit (1). Advances in the understanding of MPM tumor biology have led to the development of multiple novel targeted agents currently in preclinical and clinical development. Many of these therapies lack a biomarker for activity, and results so far have not delivered an effective clinical therapy (2).

A molecular target of significant interest in MPM is BRCA1-associated protein 1 (BAP1) (3, 4, 5). BAP1 mutations are frequent in MPM (23–67%) and in other tumor types, including uveal melanoma (31–50%), cholangiocarcinoma (20–25%), and clear cell renal cell carcinoma (CCRCC) (8–14%) (6, 7, 8, 9, 10, 11, 12, 13, 14, 15, 16, 17, 18, 19). BAP1 is a deubiquitinase (DUB) that binds to a number of transcription factors through which it regulates gene transcription and modulates cellular pathways, such as DNA repair, cell cycle, and cell death (4, 5). The response to drugs that act upon these pathways, including poly(ADP-ribose) polymerase (PARP) and enhancer of zeste homolog 2 inhibitors, has been shown to be increased in the absence of BAP1 function (20). Clinical trials of these drugs in BAP1-mutant MPM are underway (21). In addition to its function as a nuclear DUB, a recent report suggests that BAP1 also has cytoplasmic functions involving the regulation of cell death and mitochondrial metabolism (22).

We have previously demonstrated that loss of BAP1 function results in sensitivity to the death receptor (DR) agonist recombinant tumor necrosis factor (TNF)–related apoptosis–inducing ligand (rTRAIL) (23). TRAIL is a member of the TNF cytokine superfamily. It activates the extrinsic apoptotic pathway by binding to either of two DRs, DR4 or DR5, which leads to the recruitment of the adaptor protein Fas-associated protein with death domain and caspase-8 to form the death-inducing signaling complex (24). Once formed, catalytic subunits of caspase-8 are cleaved and activate downstream effector caspases triggering apoptosis (25, 26). Activation of this pathway by TRAIL is specific to cancer cells; however, the mechanism of this selectivity is poorly understood (27, 28). Several therapeutic DR agonists including rTRAIL and agonistic DR4/5 antibodies have been developed (29, 30, 31). Clinical trials of such agents to date have demonstrated broad tolerability but unfortunately limited therapeutic benefit (32). Potential reasons include the suboptimal pharmacokinetics of compounds, resistant cell populations, and the lack of a targeting biomarker (33). Novel DR agonists with improved pharmacokinetics are in development, and potential biomarkers such as BAP1 are emerging (34, 35).

We have extensively validated the association between loss of BAP1 function and increased sensitivity to rTRAIL in *in vitro*, *in vivo*, and *ex vivo* models (23). Here, we set out to delineate the mechanisms underlying this association. We hypothesize that BAP1 activity modulates expression of proteins of the extrinsic and intrinsic apoptosis pathways with an increase in proapoptotic protein expression in the absence of BAP1 activity. We demonstrate that both BAP1 activity and rTRAIL sensitivity correlate with expression of the DRs, DR4 and DR5, at the transcriptional level. As BAP1 lacks DNA-binding sites, we searched for the transcriptional factor that cooperates with BAP1 to modulate expression of DR4 and DR5 identifying the polycomb group protein Ying Yang 1 (YY1).

## Results

### Loss of BAP1 activity correlates with increased DR4 and DR5 expression and increased rTRAIL sensitivity

**Figure 1:**
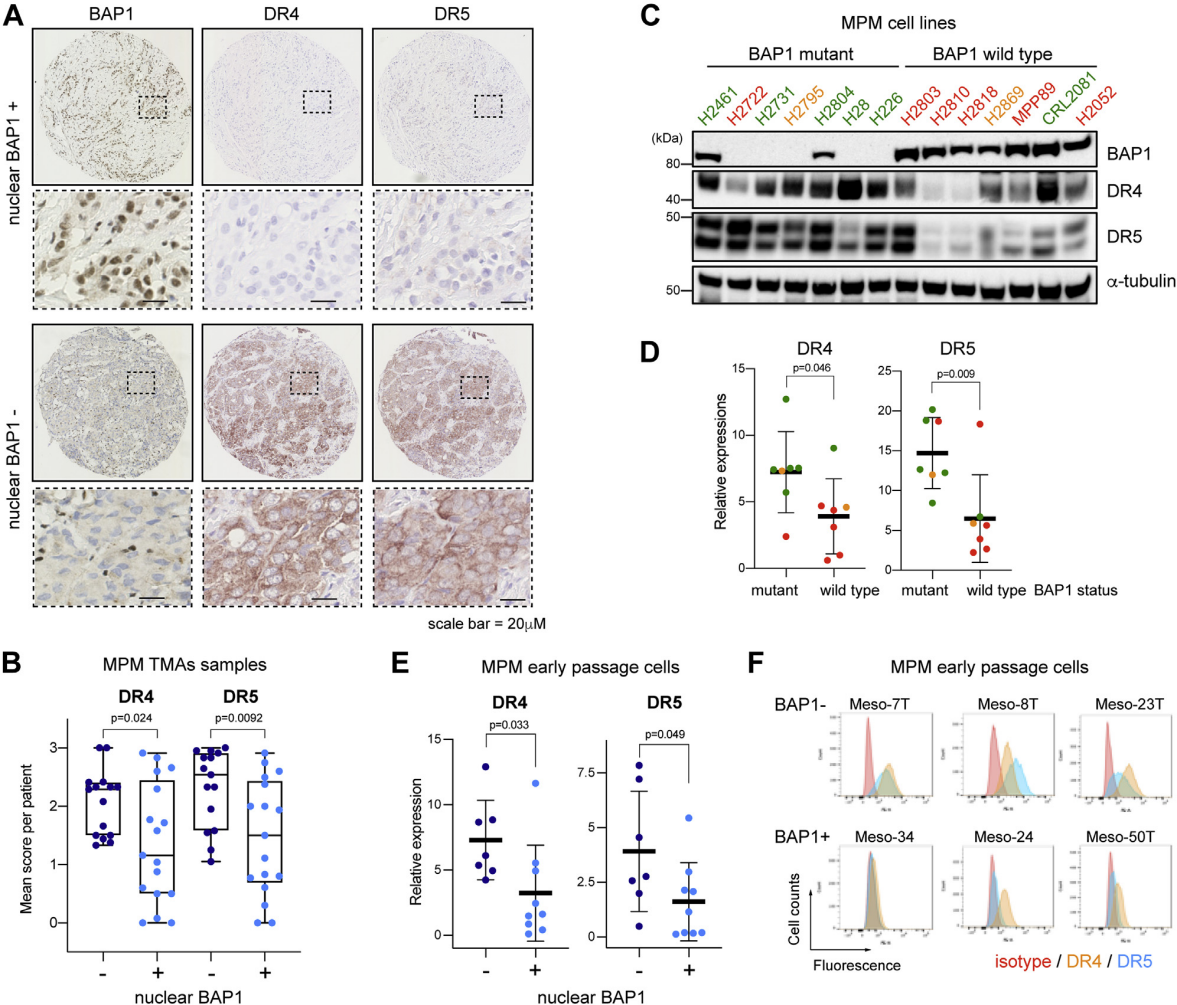
**Expression levels of DR4 and DR5 are inversely correlated with BAP1 expression in malignant pleural mesothelioma.***A*, representative images of immunohistochemistry of DR4 and DR5 in a core from an MPM tissue microarray (TMA) with or without nuclear BAP1 expression (from 88 cores of 32 patients). *B*, semiquantitative analysis of DR4 and DR5 expression in MPM TMA cores with (n = 42) and without (n = 46) nuclear BAP1 expression. Each *dot* represents an average score per patient (n = 32). *t* Test; *p* = 0.024 (DR4) and *p* = 0.0092 (DR5). See Experimental procedures section for details. *C*, immunoblots of DR4, DR5, and BAP1 protein expression in BAP1-mutant (n = 7) *versus* wt-BAP1 (n = 7) MPM cell lines. Duplet bands of DR5 represent two isoforms, DR5-short (DR5-S) and DR5-long (DR5-L). Sensitivity to rTRAIL treatment is indicated as font color: *green* sensitive (S); *orange* partially sensitive (PS); and *red* resistant (R). *D*, quantitative analysis of immunoblot intensity of DR4 and DR5 in wt BAP1 and BAP1-mutant MPM cell lines (DR4 *t* test, *p* = 0.046; DR5 *t* test, *p* = 0.009). *Dot color* indicates the sensitivity to rTRAIL treatment as shown in (*C*). *E*, quantitative analysis of immunoblot intensity of DR4 and DR5 in early passage MPM cells with (+) and without (−) nuclear BAP1 expression. (DR4 *t* test, *p* = 0.033; DR5 *t* test, *p* = 0.049). *F*, flow cytometry analysis of DR4 and DR5 cell surface expression in early passage MPM cells with (BAP1+) and without (BAP1−) nuclear BAP1 expression alongside an isotype control. BAP1, BRCA1-associated protein 1; DR, death receptor; MPM, malignant pleural mesothelioma; rTRAIL, recombinant tumor necrosis factor–related apoptosis-inducing ligand.

We have previously shown that MPM cells with loss of BAP1 function are more sensitive to treatment with rTRAIL (23). To determine the mechanism underlying this, we investigated the expression of DRs, DR4 and DR5, the levels of which are known to significantly contribute to TRAIL response (36, 37), and nuclear BAP1 expression, a surrogate for wt-BAP1 status (7). Immunohistochemical analysis of human tissue microarrays (TMAs) (88 cores from 32 patients) (Fig. 1*A*) demonstrated a significant correlation between loss of nuclear BAP1 expression and higher DR4 and DR5 expression (Figs. 1*B* and S1). This correlation was further supported by immunohistochemistry in primary MPM tissue samples collected as part of the MSO1 clinical trial (NCT00075699) (38); samples that lacked nuclear BAP1 also showed elevated levels of DR4 and DR5 (Fig. S2, *B* and *C*). Interestingly, when we used antibodies against cytokeratin 5 (CK5) and calretinin to confirm the areas of mesothelioma, we observed higher expression of DR4 and DR5, where CK5 or calretinin is expressed. This suggests that DR4 and DR5 are expressed in mesothelioma cells but not in surrounding stromal tissue (Fig. S2*A*). The expression of DRs on cancer cells but not nontransformed cells, including stromal tissue, is an existing theory for the selectivity of rTRAIL and other DR agonists for cancer cells, which our data support (36).

We further confirmed the correlation of loss of BAP1 activity and high DR4 and DR5 expression in a panel of MPM cell lines. Immunoblot analysis of MPM cell lines (seven BAP1 mutants and seven wt-BAP1) overall demonstrated a higher level of DR4 and DR5 expression in BAP1-mutant *versus* wt-BAP1 cell lines (Fig. 1, *C* and *D*). The wt-BAP1 cell line, CRL2081, however expressed a high level of DR4 and was found to be rTRAIL sensitive. The wt-BAP1 cell line H2803 expressed a high level of DR5, yet remained rTRAIL resistant. It cannot simply be inferred, therefore, that expression levels of DR4 or DR5 alone determine rTRAIL sensitivity in these wt-BAP1 cells. Indeed, the apoptotic pathway consists of dozens of proteins, many of which are mutated in cancer cells. We hypothesize that it is the balance of proapoptotic and antiapoptotic factors that determine TRAIL sensitivity, of which DR4 and DR5 are likely to be dominant but not fully determinant. Indeed, we observed additional heterogeneous changes in expression of 20 other proteins involved in the extrinsic and intrinsic apoptosis pathways; however, they did not directly correlate with the mutational status of BAP1 or rTRAIL sensitivity (Fig. S3).

We have previously shown that strong nuclear BAP1 expression is highly correlated with rTRAIL resistance in human early passage and unsequenced MPM cultures (MesobanK UK) (23, 39, 40, 41). Here, in further support of a correlation between loss of BAP1 activity and increased DR4 and DR5 expression and rTRAIL sensitivity, immunoblot analysis revealed that DR4 and DR5 expression was higher in MPM cultures with loss of nuclear BAP1 expression, and these cells were more sensitive to rTRAIL treatment (Figs. 1*E* and S4). Flow cytometry analysis also showed higher surface expression of DR4 and DR5 in MPM cultures with loss of nuclear BAP1 expression (Fig. 1*F*). Taken together, our data demonstrate strong inverse correlations between BAP1 expression and DR4 and DR5 expression, which may underlie the ability of BAP1 to determine rTRAIL sensitivity.

TRAIL has been documented in some cells to induce antiapoptotic, rather than proapoptotic, pathways. Therefore, we investigated expression of antiapoptotic proteins following treatment with rTRAIL (42, 43, 44, 45, 46). We examined cellular FLICE-like inhibitory protein, a catalytically inactive caspase-8 homolog that competes with caspase-8, inhibitors of apoptosis proteins (cellular inhibitor of apoptosis 1 and 2), mitogen-activated protein kinase, and NFκB pathways that enhance proliferation and induce cellular inhibitor of apoptosis proteins (36). We saw no induction of these proteins, excluding this as a mechanism of TRAIL resistance in BAP1-mutant cells (Fig. S5).

### Loss of BAP1 function increases DR4 and DR5 expression in malignant but not in nontransformed cells

**Figure 2:**
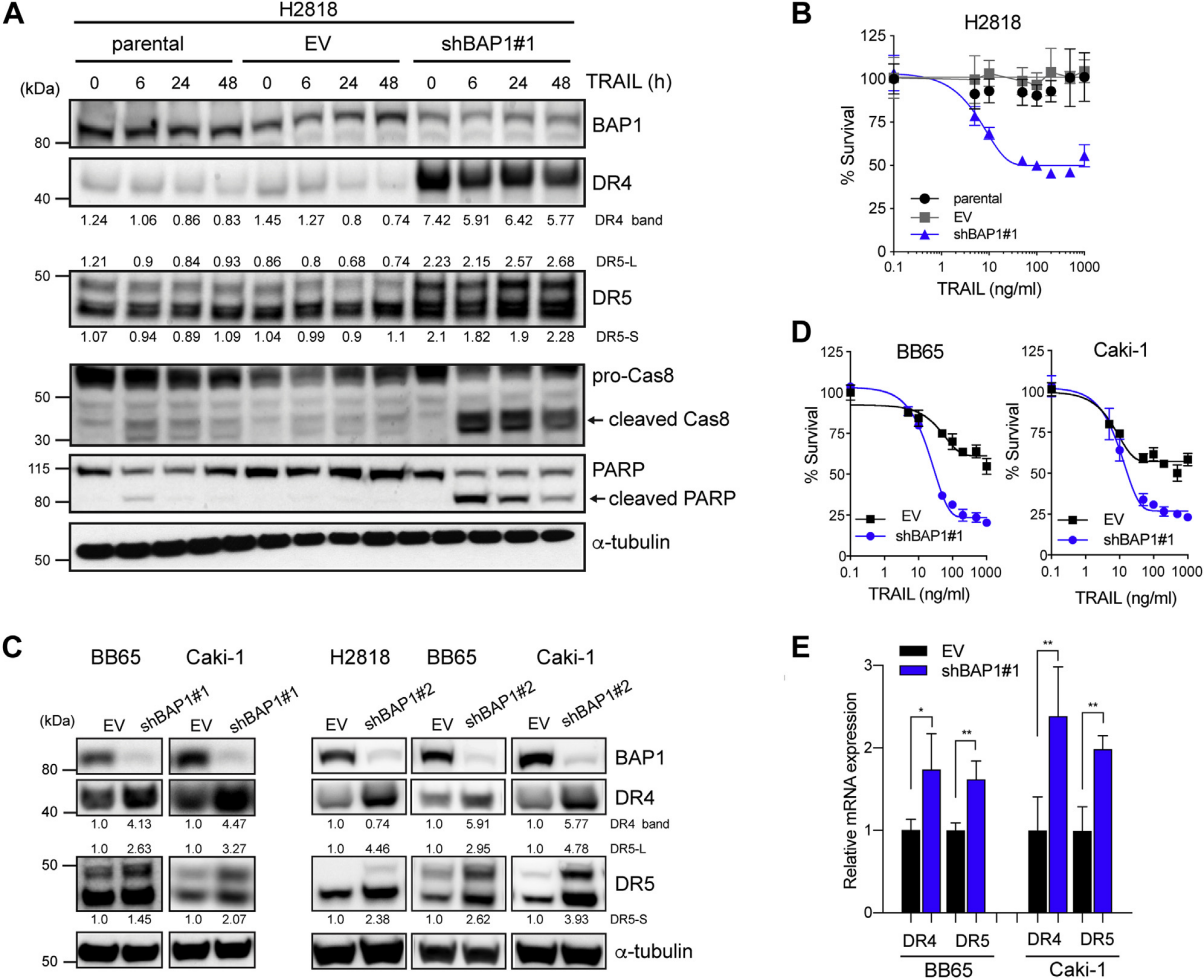
**BAP1 knockdown increases death receptor expression and TRAIL sensitivity in cancer cells.***A*, immunoblots of proapoptotic proteins in parental, BAP1 shRNA (shBAP1—clone 1) or empty vector (EV) shRNA transduced BAP1-wt MPM cells (H2818) across multiple time points (0, 6, 12, 24, and 48 h) post rTRAIL treatment (100 ng/ml). Duplet bands of DR5 represent two isoforms, DR5-short (DR5-S) and DR5-long (DR5-L). The bands were quantified and normalized to an average of parental cells data. *B*, cell viability assay of parental, shBAP1-transduced, or EV-transduced H2818 cells following treatment with a dose range of rTRAIL (0–1000 ng/ml) for 72 h. *C*, immunoblot analysis in BAP1-wt-clear cell renal cell carcinoma (CCRCC) cells (BB65 and Caki-1) and MPM cells (H2818) transduced with BAP1 (shBAP1 1 or shBAP1 2) or EV shRNA. The bands were quantified and normalized to EV. *D*, cell viability assay of EV-transduced or shBAP1-transduced wt-BAP1 CCRCC cells following treatment with a dose range of rTRAIL (0–1000 ng/ml) for 72 h. *E*, relative expression of DR4 and DR5 mRNA in CCRCC cells transduced with EV or shBAP1 assessed by quantitative PCR. Relative mRNA expression was normalized to beta-2-microgloblin (B2M) expression. Data shown are the mean ± SD of two experiments performed in triplicates. *t* test; ∗*p* < 0.05, ∗∗*p* < 0.01. BAP1, BRCA1-associated protein 1; MPM, malignant pleural mesothelioma; rTRAIL, recombinant tumor necrosis factor–related apoptosis-inducing ligand; TRAIL, tumor necrosis factor–related apoptosis-inducing ligand.

To further investigate the relationship between BAP1 and DR4 and DR5 expression, we knocked down BAP1 expression in a BAP1-wt MPM cell line using lentiviral shRNA constructs. BAP1 knockdown significantly increased expression of both DR4 and DR5 (Fig. 2*A*). DR5 has two isoforms; the expression of both was found to increase with BAP1 knockdown (Fig. 2*A*). It is not understood if there is a difference in function between these isoforms (47, 48). We confirmed that BAP1 knockdown resulted in increased sensitivity to rTRAIL in these cells (Fig. 2*B*). Induction of cleaved caspase-8 and cleaved PARP was observed only in BAP1 knockdown cells indicating apoptosis activation only in the absence of BAP1. To examine the effect across additional tumor types, we next knocked down BAP1 in two wt-BAP1 CCRCC cell lines. This also resulted in increased expression of DR4 and DR5 and increased sensitivity to rTRAIL (Fig. 2, *C* and *D*). An additional shRNA clone confirmed these results in these two CCRCC lines and in a wt-BAP1 MPM cell line (Figs. 2*C* and S6). BAP1 knockdown also lead to increased DR4 and DR5 mRNA levels (Fig. 2*E*) indicating that the effect of BAP1 on DR4 and DR5 expression is at the transcriptional level. Significantly, BAP1 knockdown in human lung fibroblasts and human bronchial epithelial cells (HBECs) did not affect expression of DR4 and DR5 or sensitivity to rTRAIL suggesting this effect is specific to malignant cells (Fig. S7).

### BAP1 negatively regulates transcription of *DR4* and *DR5*

**Figure 3:**
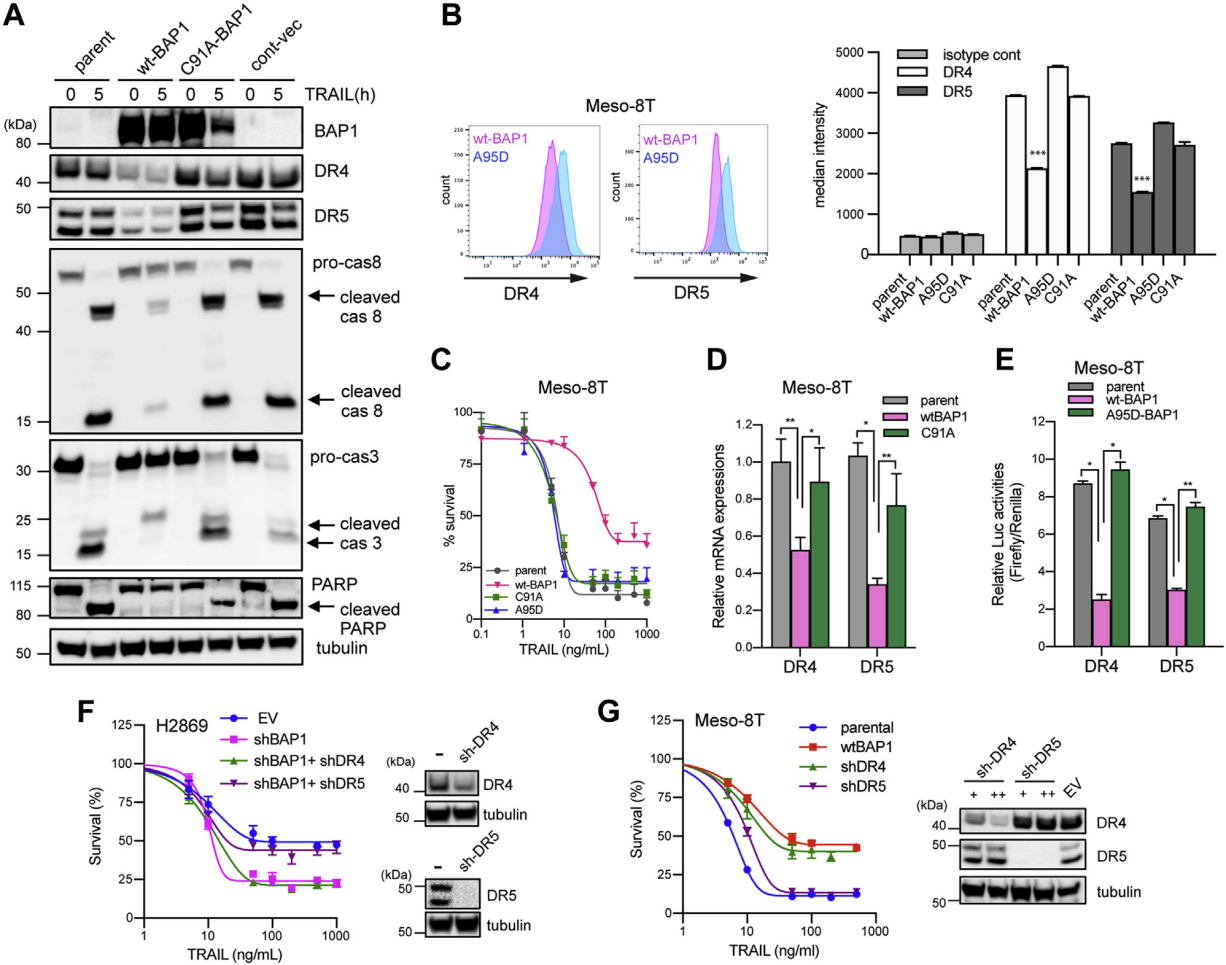
**The deubiquitinase (DUB) function of BAP1 regulates the transcription of DR4 and DR5.***A*, immunoblots of proapoptotic proteins in BAP1 null early passage mesothelioma cells (Meso-8T) transduced with constructs expressing wt-BAP1 (wt-BAP1), DUB-mutant BAP1 (C91A-BAP1), or a control vector (cont-vec) untreated and after 5 h of rTRAIL treatment (50 ng/ml). *B*, flow cytometry analysis of cell surface expression of DR4 and DR5 in Meso-8T cells transduced with constructs expressing wt-BAP1 (wt-BAP1) or one of two DUB-mutant BAP1 vectors (C91A or A95D). One-way ANOVA; ∗∗∗*p* < 0.001. *C*, cell viability assay of Meso-8T cells transduced with wt-BAP1 or one of two DUB-mutant BAP1 vectors (C91A or A95D) following treatment with a dose range of rTRAIL (0–1000 ng/ml) for 72 h. *D*, relative DR4 and DR5 mRNA expression in parental Meso-8T cells and cells transduced with wt-BAP1 or C91A-BAP1. Relative mRNA expression was normalized to beta-2-microgloblin (B2M) expression. Data are shown as the mean ± SD of two experiments performed in triplicates. ∗*p* < 0.05; ∗∗*p* < 0.01. *E*, reporter assay for promoter activities of DR4 and DR5 in parental Meso-8T cells transduced with a luciferase reporter under the control of DR4 or DR5 promoter and cells further transduced with wt-BAP1 or A95D-BAP1. Firefly luciferase/Renilla luciferase ratios were determined as relative luciferase activities. Data are shown as the mean ± SD of two experiments (n = 6 in each experiment). ∗*p* < 0.05; ∗∗*p* < 0.01. *F*, cell viability assay of wt-BAP1 H2869 cells transduced with empty vector (EV) or shBAP1 following treatment with a dose range of rTRAIL (0–1000 ng/ml) for 72 h and for shBAP1 cells further transduced with DR4 (shDR4) or DR5 shRNA (shDR5) following the same treatment. *G*, cell viability assay of parental BAP1 null Meso-8T early passage MPM cells transduced with wt BAP1 (wt-BAP1) or DR4 (shDR4) or DR5 shRNA (shDR5) following treatment with a dose range of rTRAIL (0–1000 ng/ml) for 72 h. +, ++; lentiviral titer. Error bars represent the SD. BAP1, BRCA1-associated protein 1; rTRAIL, recombinant tumor necrosis factor–related apoptosis-inducing ligand.

To test if BAP1 DUB activity is required for transcriptional regulation of DR4 and DR5 expression, we next transduced a BAP1-null early passage MPM cell line, Meso-8T, with a lentiviral construct expressing wt-BAP1 or BAP1 with an inactivating mutation in the DUB site C91A-BAP1 or A95D-BAP1. Various mutations at C91 have been reported and shown in COSMIC (https://cancer.sanger.ac.uk/cosmic) (49). A95D is a naturally occurring mutation in MPM tumors in patients (6). Transduction with wt-BAP1 but not C91A-BAP1 resulted in a decrease in DR4 and DR5 expression (Fig. 3*A*). Flow cytometry confirmed a decrease in surface expression of DR4 and DR5 in cells transduced with wt-BAP1 but not C91A-BAP1 or A95D-BAP1 (Fig. 3*B*). Cell survival assays confirmed that transduction with wt-BAP1, but not C91A-BAP1 or A95D-BAP1, resulted in a significant reduction in rTRAIL sensitivity (Fig. 3*C*). Concordantly, we saw decreased activation of caspase-8, caspase-3, and reduced PARP cleavage in wt-BAP1-transduced cells relative to C91A-BAP1-transduced cells when treated with rTRAIL (Fig. 3*A*) reflective of reduced activation of the extrinsic apoptotic pathway in the presence of wt-BAP1. Quantitative PCR (qPCR) analysis demonstrated that DR4 and DR5 mRNA expressions were both decreased in cells transduced with wt-BAP1 relative to those transduced with C91A-BAP1 suggesting that regulation of DR4 and DR5 expression by catalytically active BAP1 is at the transcriptional level (Fig. 3*D*). These results were confirmed in a further MPM cell line, H28, which harbors a BAP1 splice site mutation commonly found in MPM tumors (Fig. S8) (6). We have also previously confirmed reduced DR4 and DR5 expression in C91A BAP1–transduced relative cells to BAP1-wt–transduced H226 cells using flow cytometry analysis (23). Subsequently, we tested the effect of BAP1 on *DR4* and *DR5* transcription more directly. Meso-8T cells were transduced with lentiviral vectors with luciferase reporters under the control of *DR4* or *DR5* promoters (50). These reporter cells were also transduced with either wt-BAP1 or A95D-BAP1. Cells transduced with wt-BAP1 displayed a significantly lower luciferase activity than those transduced with A95D-BAP1 or the parental cell line reflecting decreased *DR4* and *DR5* transcriptional activity in the presence of functional BAP1 (Fig. 3*E*).

Together, the aforementioned results support that the DUB activity of BAP1 mediates transcriptional repression of *DR4* and *DR5*. To test whether this in turn determines rTRAIL sensitivity, we used two complementary approaches. First, we knocked down *DR4* or *DR5* in wt-BAP1 H2869 MPM cells transduced with BAP1 shRNA. BAP1 knockdown increased the sensitivity of H2869 cells to rTRAIL as expected (Fig. 3*F*). Interestingly, DR5, but not DR4 knockdown, in shBAP1-H2869 cells abolished the effect of BAP1 knockdown, resulting in rTRAIL resistance (Fig. 3*F*). Second, we knocked down *DR4* or *DR5* in the BAP1-null, rTRAIL-sensitive Meso-8T cell line. *DR5* knockdown only slightly decreased rTRAIL sensitivity, but *DR4* knockdown reduced it to a similar level as transduction with wt BAP1 (Fig. 3*G*). These data are in line with previous reports showing preferential use of one of the two receptors by distinct cell types (31). For example, hematological cancers seem to prefer DR4 for induction of apoptosis (51, 52), whereas solid tumors appear to exhibit heterogeneity in DR preference (31, 53, 54).

### YY1 negatively regulates transcription of *DR4* and *DR5*

**Figure 4:**
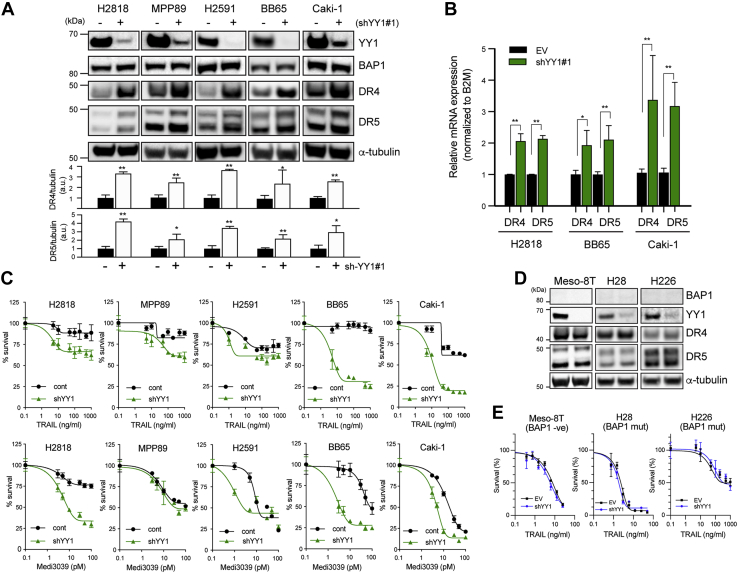
**YY1 knockdown increases the expression of DRs and rTRAIL-induced cell death.***A*, immunoblot analysis in BAP1-wt MPM cells (H2818, MPP89, and H2591) or CCRCC cells (BB65 and Caki-1) transduced with YY1 shRNA-clone 1 (+) or an empty vector (EV) shRNA (−). Quantitative analysis of DR4 and DR5 bands from three independent experiments was performed. Average data after normalization to tubulin were shown as bar graphs. *B*, relative DR4 and DR5 mRNA expression in MPM cells (H2818) and CCRCC cells (Caki-1 and BB65) transduced with YY1-shRNA or EV-shRNA. Relative mRNA expression was normalized to beta-2-microgloblin (B2M) expression. Data are shown as the mean ± SD of two experiments performed in triplicates. ∗*p* < 0.05; ∗∗*p* < 0.01. *C*, cell viability assays of BAP1-wt MPM and CCRCC cells transduced with EV shRNA or shYY1 (clone 1) following treatment with a dose range of rTRAIL (0–1000 ng/ml) or MEDI3039 (0.1–100 pM) for 72 h. Error bars represent the SD. *D*, immunoblot analysis in BAP1-null early passage MPM Meso-8T cells and BAP1-mutant MPM cell lines (H28 and H226) transduced with YY1 (clone 1) or EV shRNA. *E*, cell viability assay of EV-transduced or shYY1-transduced (clone 1) cells described in *D*, following treatment with a dose range of rTRAIL (0–1000 ng/ml) for 72 h. BAP1, BRCA1-associated protein 1; CCRCC, clear cell renal cell carcinoma; DR, death receptor; MPM, malignant pleural mesothelioma; rTRAIL, recombinant tumor necrosis factor–related apoptosis-inducing ligand; YY1, Ying Yang 1.

As BAP1 does not bind to DNA directly (5), we aimed to identify transcription factors that bind to the promoter regions of *DR4* and *DR5*. Bioinformatics analysis of 2000 nucleotides of the promoter region of *DR4* and *DR5* was conducted. From candidates identified (Fig. S9), YY1 was selected for further analysis as it has previously been shown to negatively regulate *DR5* expression in prostate cancer (55, 56). Furthermore, YY1 has been shown to bind directly to BAP1, with the C-terminal region of BAP1 essential for this interaction, forming a complex capable of regulating gene expression (57). *YY1* knockdown with two different shRNA clones in wt-BAP1 MPM and CCRCC cells resulted in increased expression of both DR4 and DR5 without affecting steady-state levels of BAP1 (Figs. 4*A* and S10). shRNA knockdown of BAP1 in MPM cells also did not affect steady-state levels of YY1 (Fig. S11*A*). In addition, we did not observe any difference in YY1 expression based on BAP1 mutational status and BAP1 expression level (Fig. S11*B*). qPCR analysis confirmed increased mRNA expression of *DR4* and *DR5* in cells transduced with YY1 shRNA (Fig. 4*B*). *YY1* knockdown also significantly increased sensitivity to rTRAIL and the DR5 agonist Medi3039 in MPM and CCRCC cells (Fig. 4*C*) (58). We also determined if YY1 is able to regulate DR4 and DR5 expression in the absence of BAP1. We knocked down YY1 in BAP1-mutant MPM cell lines and BAP1-null early passage MPM cells and assessed the expression of DR4 and DR5 and rTRAIL sensitivity. Neither DR4/DR5 expression nor TRAIL sensitivity increased in the YY1 knockdown cells (Fig. 4, *D* and *E*) in these BAP1-mutant cells, unlike in BAP1-wt cells, suggesting that BAP1 is required for *DR4/5* regulation by YY1. These data demonstrate that YY1, in addition to BAP1, modulates expression of DR4 and DR5. As YY1 and BAP1 have been shown to form a complex capable of regulating gene expression, it is likely that this complex regulates *DR4* and *DR5* expression (57).

### BAP1 and YY1 act at DR4 and DR5 promoters to facilitate transcriptional repression

**Figure 5:**
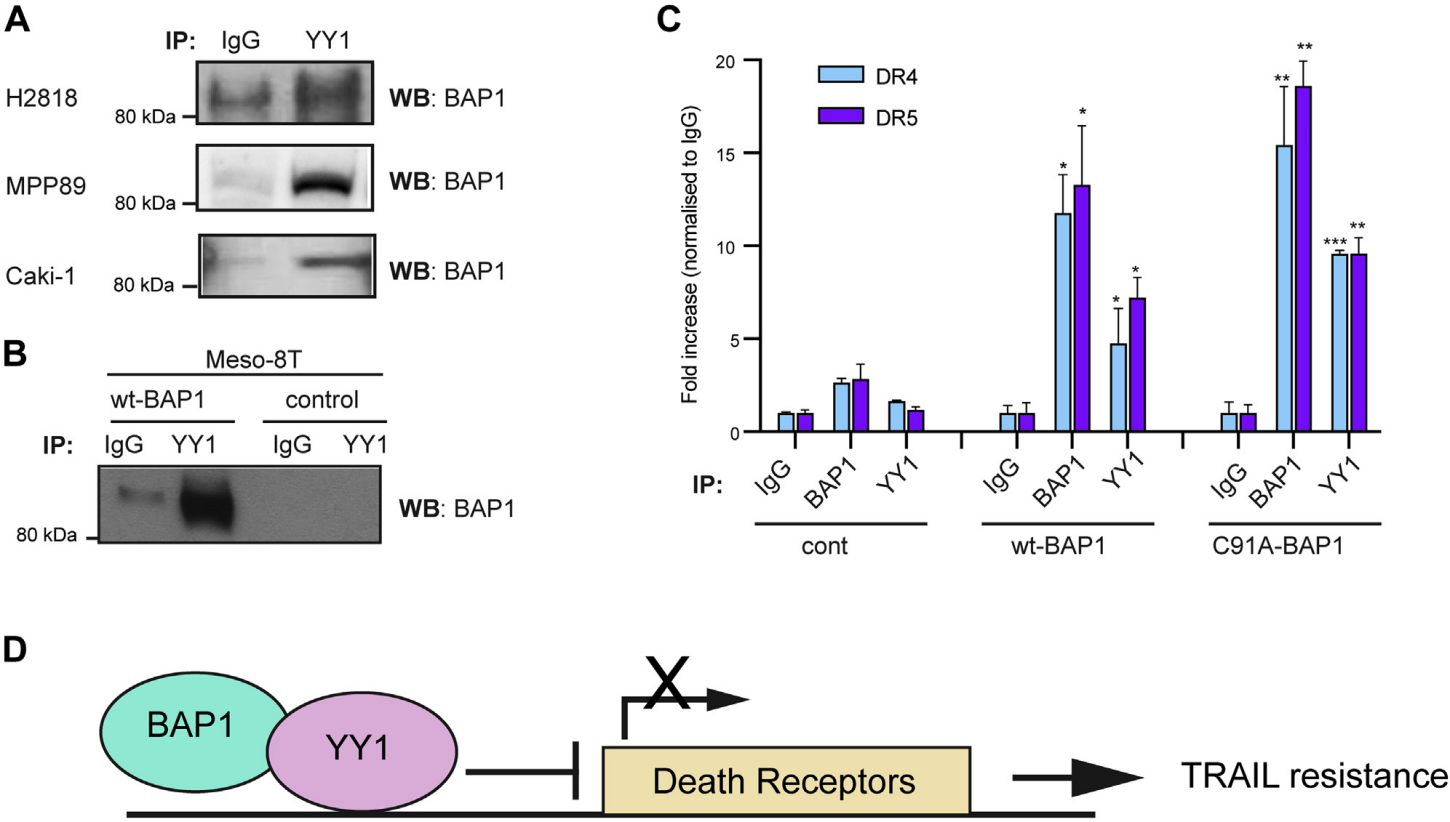
**YY1 recruits BAP1 to the promoter regions of DR4 and DR5 and represses their transcriptional activities.***A*, co-immunoprecipitation (co-IP) of endogenous YY1 and BAP1 in MPM (H2818 and MPP89) and CCRCC (Caki-1) cells. *B*, co-IP of YY1 and BAP1 in BAP1 null early passage MPM Meso-8T cells transduced with wt BAP1 (wt-BAP1) or a control vector. *C*, enrichment of BAP1 and YY1 in the promoter regions of DR4 and DR5. Meso-8T cells were overexpressed with wt-BAP1, catalytically inactive BAP1-mutant (C91A-BAP1) or a control vector (cont). Chromatin immunoprecipitation (ChIP) was performed against BAP1, YY1, or IgG control followed by quantitative PCR using primers specific for promoter regions of DR4 or DR5. Error bars represent the SD. *p* Values are calculated to compare against IgG control using Student's *t* test (n = 3); ∗*p* < 0.05, ∗∗*p* < 0.01. *D*, schematic model of the transcriptional regulation of TRAIL DRs by BAP1 and YY1. BAP1, BRCA1-associated protein 1; CCRCC, clear cell renal cell carcinoma; DR, death receptor; MPM, malignant pleural mesothelioma; TRAIL, tumor necrosis factor–related apoptosis-inducing ligand; YY1, Ying Yang 1.

BAP1 has been shown to form a ternary complex with YY1 and host cell factor 1 (HCF-1) in HeLa cells (57). Through its coiled-coil motif, BAP1 directly interacts with the zinc fingers of YY1, whereas HCF-1 interacts with the middle region of YY1 and is essential for the formation of the ternary complex *in vivo* (57). Therefore, we aimed to determine if BAP1 and YY1 also interact directly in MPM and CCRCC cells. Protein extracts from H2818, MPP89, and Caki-1 cells were co-immunoprecipitated using anti-YY1 antibody or immunoglobulin G (IgG) as a control. Immunoblot confirmed the interaction of endogenous YY1 with BAP1 (Fig. 5*A*). To verify the specificity of these results, we compared results of co-immunoprecipitation (co-IP) assay in BAP1-null MPM cells (Meso-8T) that were transduced with wt-BAP1 or a control vector alone. A strong interaction of YY1 and BAP1 was detected only in cells transduced with wt-BAP1 but not the control vector, confirming the specificity of the YY1–BAP1 interaction (Fig. 5*B*). Here, co-IP assay demonstrates that YY1 also interacts with HCF-1 in wt-BAP1 CCRCC cells (Fig. S12). However, we have previously shown that MPM cells expressing BAP1 that lacks the binding domain for HCF-1 are not significantly different in their TRAIL sensitivity compared with cells expressing wt-BAP1 (23). This suggests that the HCF-1/BAP1 interaction does not determine TRAIL sensitivity and is unlikely to be involved in DR regulation.

In addition to physical interactions, we sought to examine the functional interaction between YY1 and BAP1. As BAP1 does not have a DNA-binding domain, but directly interacts with the transcriptional repressor YY1, we hypothesized that BAP1 and YY1 are recruited to the promoter regions of DR4 and DR5. Chromatin immunoprecipitation (ChIP) assays were performed with antibodies for BAP1, YY1, or IgG as a control. The immunoprecipitated DNA was analyzed with probes for DR4 or DR5 by qPCR in Meso-8T cells transduced with wt-BAP1, C91A-BAP1, or a control vector. Both BAP1 and YY1 were enriched in the promoter regions of DR4 and DR5 in cells transduced with wt-BAP1 but not the control vector (Fig. 5*C*). Interestingly, BAP1 and YY1 were also enriched in these promoter regions in cells transduced with C91A-BAP1 indicating BAP1 and YY1 are recruited to these promoter regions regardless of DUB activity. This finding is consistent with previous reports that catalytically inactive BAP1 is also recruited to FoxK2-binding regions (59). Catalytically inactive BAP1 has also previously been shown to form a complex with YY1 (57). Taken together, we show that BAP1 and YY1 are recruited at the promoters of TRAIL receptors and are necessary to initiate transcriptional regulation of TRAIL receptors.

## Discussion

We have recently reported that loss of BAP1 function is a predictive biomarker for rTRAIL sensitivity in cancer (23). In this study, we delineate the underlying molecular mechanism. We demonstrate BAP1 and the transcriptional regulator YY1 act at the promoter regions of *DR4* and *DR5*, where they facilitate transcriptional repression of *DR4* and *DR5*, which requires BAP1 DUB activity. Decreased cell surface expression of DR4 and DR5 and reduced activation of the apoptotic pathway in turn mediates rTRAIL resistance in wt-BAP1 cells. Conversely, increased cell surface expression of DR4 and DR5 in BAP1-mutant cells mediates the observed increased sensitivity to rTRAIL. Various mechanisms of resistance to rTRAIL and other DR agonists have been suggested (36). Evidence supports that low expression of DR4 and DR5 because of mutations, promoter methylation, constitutive endocytosis, or deficient transport to the cell surface is important (36, 60, 61, 62). Indeed, strategies to enhance the efficacy of rTRAIL treatment, such as a combination with chemotherapeutic drugs, have been demonstrated to mediate these effects through increased DR expression (25). Our results are consistent with these data and support the centrality of DR expression in TRAIL therapeutics.

YY1 inhibition has previously been shown to upregulate DR5 expression and enhance rTRAIL sensitivity in prostate cancer and B-non-Hodgkin's lymphoma cells (55, 56, 63). Here however, we show that YY1 is involved in the transcriptional regulation of both DR4 and DR5 and is enriched at the promoters of both DR4 and DR5 when BAP1 is present. BAP1 is known to form multiprotein complexes including as many as ten partners, which in turn determine the precise targets of its DUB activity (5, 64). It has previously shown that BAP1 forms a multiprotein complex with YY1 and the transcriptional cofactor HCF-1 (57). Although not investigated here, further work might identify additional cofactor(s) that direct BAP1 and YY1 to the DR4 and DR5 promoters. We have previously shown that mutation of the additional sex combs-like (ASXL)-binding site on BAP1 and ASXL1 knockdown also increases rTRAIL sensitivity (23). The BAP1–ASXL1 complex is a polycomb repressor DUB complex capable of deubiquitination of histone 2A at lysine 119 (H2A119Ub), a process that modulates expression of the polycomb genes (5). Interestingly, YY1 has also been shown to interact with polycomb proteins (57, 65). It may therefore be that YY1 interacts with both BAP1 and ASXL1 to modulate DR expression through the deubiquitination of H2A119Ub or that BAP1 and YY1 form an alternate complex with a different target that modulates histone and chromatin structure DR expression.

YY1 and BAP1 may be involved more widely in the transcriptional regulation of the TNF receptor superfamily. Nitric oxide has been shown to inhibit YY1 binding to the *Fas* promoter resulting in Fas upregulation and cell sensitization to Fas ligand–induced apoptosis in prostate cancer (66). YY1 has also been shown to suppress the Fas promoter activity in B-non-Hodgkin's lymphoma and colon cancer (67, 68). We have also previously demonstrated that BAP1 knockdown sensitizes MPM cells to Fas ligand and TNF alpha (23).

Although BAP1 was originally identified as a tumor suppressor gene, accumulating evidence has revealed roles in multiple clinically targetable pathways (69, 70, 71, 72). Indeed, we have proposed BAP1 expression to be a stratifying biomarker for sensitivity to DR agonists (23), and our work here provides a biological rationale for this. The current study also demonstrates that YY1 knockdown enhances the sensitivity to TRAIL and DR5 agonist. YY1 is overexpressed in many types of cancer, and high expression correlates with poor clinical outcomes and resistance to chemotherapy and immunotherapy making it an attractive therapeutic target (73, 74). Thus, targeting the BAP1–YY1 axis may be an additional novel therapeutic strategy in TRAIL therapeutics.

## Experimental procedures

### Cell culture

All cancer cell lines were obtained from the Wellcome Trust Sanger Institute except the H226 line that was kindly gifted by Dr P. Szlosarek (Barts Cancer Institute). Cancer cell lines were cultured in RPMI1640, Dulbecco's modified Eagle's medium (DMEM), or DMEM and nutrient mix 12 medium (DMEM:F12) supplemented with 10% fetal bovine serum (FBS), penicillin/streptavidin, and sodium pyruvate. Early passage human mesothelioma cells were obtained from MesobanK UK (39) and cultured in RPMI1640 medium supplemented with 5% FBS, 25 mM Hepes, penicillin/streptavidin, and sodium pyruvate. Primary human lung fibroblasts (kind gift from Dr R. Chambers at University College London) were cultured in DMEM media supplemented with 10% FBS and penicillin/streptavidin in an incubator with 10% CO_2_ (75). Experiments were conducted on cells between passage 6 and 8. Primary HBECs were obtained from endobronchial biopsies with patient consent as previously described (76). Ethical approval was obtained through the National Research Ethic Committee (reference 06/Q0505/12). All studies involving human subjects abide by the Declaration of Helsinki principles. HBECs were cultured in bronchial epithelial growth medium (Lonza) on top of 3T3-J2 mouse embryonic fibroblast feeder cells inactivated by mitomycin-C treatment (0.4 μg/ml; Sigma–Aldrich).

### 2,3-bis-(2-methoxy-4-nitro-5-sulfophenyl)-2H-tetrazolium-5-carboxanilide cell viability assay

Cells were seeded in 96-well plates in 100 μl media per well at a density of 40,000 cells/ml 1 day prior to treatment with soluble rTRAIL (PeproTech) or MEDI3039 (MedImmune). 2,3-bis-(2-methoxy-4-nitro-5-sulfophenyl)-2H-tetrazolium-5-carboxanilide reagent and the activation solution (AppliChem, LLC and Akron Biotech) were mixed and added to the cells at the end of treatment. The plate was returned to a CO_2_ incubator to incubate for 2 h, and the absorbance at a wavelength of 490 nm was measured using a microplate reader. Relative cell viability was calculated as a fraction of viable cells relative to untreated cells.

### Immunoblotting

Cells were lysed in radioimmunoprecipitation lysis buffer (Sigma–Aldrich) with protease inhibitors (cOmplete, Mini Protease Inhibitor Cocktail; Roche) on ice to extract protein. About 30 μg of protein samples were separated by SDS-PAGE and transferred onto nitrocellulose membranes using iBlot2 Dry Blotting System (Thermo Fisher Scientific). Membranes were incubated with specific primary antibodies, washed, incubated with secondary antibodies, and visualized using an ImageQuant LAS 4000 imaging system (GE Healthcare). Anti-BAP1 antibody was purchased from Santa Cruz Biotechnology, and anti-cellular FLICE-like inhibitory protein antibody was purchased from Enzo Life Sciences. All the other antibodies were purchased from Cell Signaling Technology. Quantification of bands was performed using ImageJ (Image Processing and Analysis in Java; the National Institutes of Health).

### IP

Cells were lysed in IP buffer containing 0.2% NP-40, 20 mM Tris–HCl (pH 7.4), 150 mM NaCl, 10% glycerol, and protease inhibitors. The lysates were incubated overnight with gentle rocking with anti-YY1 antibody (ab38422; Abcam) or IgG (2729; Cell Signaling Technology). Protein-A magnetic beads (Pierce Biotechnology and Thermo Fisher Scientific) were added, and incubation was continued for 1 h. The beads were washed with IP buffer, and proteins were eluted from the beads by heating with SDS sample buffer. Proteins were separated by SDS-PAGE, and immunoblotting was performed as described previously with anti-BAP1 antibody (sc-28383; Santa Cruz).

### Plasmids

Full-length BAP1 complementary DNA was amplified by PCR from pCMV6-AC BAP1 plasmid (SC117256; Origene) and cloned into the lentiviral plasmid pCCL-CMV-flT vector. Vectors expressing BAP1-mutant constructs were generated by site-directed mutagenesis (E0554; New England Biolabs) of the pCCL-CMV-BAP1 vector as previously described (23).

### RNA interference

shRNAs were expressed as part of a mir30-based GIPZ lentiviral vector (Dharmacon). The clones used in this study include BAP1 (clone 1, V2LHS_41473; clone 2, V2LHS_41478), DR4 (V3LHS_383718), DR5 (V3LHS_328891), YY1 (clone 1, V3LHS_412955; clone 2, V3LHS_412955), and the empty GIPZ control vector.

### Lentivirus production and cell transfection

Lentiviral particles were produced by cotransfection of 293T cells with construct plasmids and the packaging plasmids pCMV-dR8.74 and pMD2.G (kind gifts from Dr Adrian Thrasher, University College London) using a DNA transfection reagent jetPEI (Source BioScience). The viral particles were concentrated by ultracentrifugation at 17,000 rpm (SW28 rotor, Optima LE80K Ultracentrifuge; Beckman) for 2 h at 4 °C. To determine the titers of prepared lentivirus, 293T cells were transduced with serial dilutions of viruses in the presence of 8 μg/ml polybrene, and protein expression was assessed by flow cytometry and immunoblotting.

### Flow cytometry

All flow cytometry analyses were performed on an LSR Fortessa analyzer (Becton Dickinson). For analysis of BAP1 expression, cells were fixed, permeabilized, and stained with primary antibody to BAP1 (1:50 dilution, SC28383; Santa Cruz) and then with an AlexaFluor 488–conjugated antimouse antibody (1:200, A-21202; Invitrogen). For analysis of surface expression of DR4 and DR5, cells were stained with 1:100 dilution of phycoerythrin-conjugated antibody (#307205 for DR4, #307405 for DR5, and #400112 for isotype; BioLegend). FlowJo (Becton Dickinson) software was used to analyze all the data.

### RT-qPCR

Total RNA was extracted from the cells using SV Total RNA Isolation System (Promega) according to the manufacturer's instructions. Complementary DNA was synthesized using iScript Reverse Transcription Supermix for RT-qPCR (Bio-Rad Laboratories). qPCR was performed using TaqMan probes (DR4: Hs00269492_m1; DR5: Hs00366278_m1; beta-2-microglobulin: Hs00187842_m1) and TaqMan Gene Expression Master Mix (Life Technologies) as per the manufacturer's protocol. Relative expression of DR4 and DR5 was calculated using comparative CT method with a reference gene, beta-2-microglobulin.

### ChIP assay

The ChIP assay was carried out using EZ ChIP Chromatin Immunoprecipitation Kit (Merck-Millipore) according to the manufacturer's instruction. Briefly, the cells were crosslinked, quenched, and lysed, and then the chromatin was fragmented by sonication shearing. Protein–DNA complexes were diluted, precleared with Protein G agarose beads, then immunoprecipitated by incubation with antibodies against BAP1 (catalog no. 78105; Cell Signaling), YY1 (catalog no. ab38422; Abcam 422) or IgG (catalog no. 2729; Cell Signaling) overnight with rotation, followed by incubation with protein G agarose beads for 1 h. After washing beads, protein–DNA complexes were eluted, reverse crosslinked to free DNA, which was then purified using spin columns and analyzed by qPCR. Primer pairs for ChIP assays were as follows: DR5; forward 5′-GGGAAGGGGAGAAGATCAAG-3′, reverse 5′-GAAGGGACCGGAACTAACCT-3′. DR4; forward 5′-CCGAATGCGAAGTTCTGTCT-3′, reverse 5′-AAGAGCCCCACACTTTGCT-3′.

### Luciferase reporter assay

Meso-8T cells were transduced with lentiviral vectors expressing a firefly luciferase reporter plasmid containing either DR4 promoter (upstream −1773/+63) or DR5 promoter (upstream −1400), plus control Renilla luciferase reporter under a control of CMV promoter (pDR4-FireflyLuc-CMV-RenillaLucDsRed2 or pDR5-FireflyLuc-CMV-RenillaLucDsRed2) vectors (50). Cells were seeded in 96-well plate, and luciferase activities were measured using Dual-Luciferase Reporter Assay System kit as described by the manufacturer (Promega). Fluc/Rluc ratios were determined as relative luciferase activities.

### Immunohistochemistry

Tumor biopsies taken from patients with MPM in the MS01 trial (NCT00075699) were stored as formalin-fixed paraffin-embedded blocks or as unstained mounted sections as previously described (38). The TMA slides containing tumor samples from patients with MPM were obtained from MesobanK UK. All studies involving human subjects abide by the Declaration of Helsinki principles. To assess expression of DR4, DR5, CK5, and calretinin, samples were first incubated in the oven at 60 °C for 30 min, then deparaffinized and rehydrated using an automated tissue processor (Tissue-Tek). Antigen retrieval was achieved by immersion in 10 mM citric acid buffer (pH 6.0) at 95 °C for 15 min. After washing with PBS and blocking with 2.5% normal goat serum, samples were incubated with primary antibody: anti-DR4 (1:500 dilution, ab8414; abcam), anti-DR5 (1:500 dilution, ab8416; abcam), anti-calretinin (1:200 dilution, NCL-L-CALRET-566; Leica Biosystems), anti-keratin 5 (BioLegend; catalog no.: 905501, 1:500 dilution) in 1% bovine serum albumin/4% serum overnight at 4 °C. Samples were incubated with ImmPRESS polymer reagent (VECTOR Laboratories) for 30 min and stained with ImmPACT Nova RED (VECTOR Laboratories). H&E staining was carried out using an automated tissue processor (Tissue-Tek). Staining for BAP1 was performed as described before using anti-BAP1 antibody (1:150 dilution, sc-28282; Santa Cruz Biotechnology) (38). Images were acquired using a NanoZoomer 2.0HT whole slide imaging system (Hamamatsu Photonics). Histology and nuclear BAP1 assessment were performed by two consultant pathologists. Intensity of DR4 and DR5 expression was assessed blindly by three independent observers and scored as follows (no staining = 0; low staining = 1; medium staining = 2; and strong staining = 3).

### Bioinformatical analysis

To identify the common transcription factors that potentially regulate these genes, the 2000 nucleotide sequence of the promoter regions of DR4 and DR5 are entered into Human Core-Promoter Finder (http://rulai.cshl.org/tools/genefinder/CPROMOTER/human.htm).

### Statistical analysis

Data were evaluated using the statistical analysis and indicated with *p* values. *p* < 0.05 was considered statistically significant. Using Prism 8 (GraphPad Software, Inc), Student's *t* test was performed to analyze differences between two groups, whereas one-way ANOVA was used to determine the differences between three or more independent groups.

For the statistical analysis of TMAs, linear mixed modeling was used to account for multiple samples per patient, including the patient ID as a random effect. Linear mixed models were implemented using the Bioconductor *Ime4* and *ImerTest* packages. Pairwise *t* test confirmed that there was no systematic bias between the score of different observers.

## Data availability

All data are contained within the article.

## Supporting information

This article contains supporting information.

## Conflict of interest

The authors declare that they have no conflicts of interest with the contents of this article.

## References

[1] Vogelzang N.J., Rusthoven J.J., Symanowski J., Denham C., Kaukel E., Ruffie P., Gatzemeier U., Boyer M., Emri S., Manegold C., Niyikiza C., Paoletti P. (2003). Phase III study of pemetrexed in combination with cisplatin *versus* cisplatin alone in patients with malignant pleural mesothelioma. J. Clin. Oncol..

[2] Nicolini F., Bocchini M., Bronte G., Delmonte A., Guidoboni M., Crinò L., Mazza M. (2020). Malignant pleural mesothelioma: State-of-the-art on current therapies and promises for the future. Front. Oncol..

[3] Schunselaar L.M., Zwart W., Baas P. (2017). Targeting BAP1: A new paradigm for mesothelioma. Lung Cancer.

[4] Wang A., Papneja A., Hyrcza M., Al-Habeeb A., Ghazarian D. (2016). BAP1: Gene of the month. J. Clin. Pathol..

[5] Carbone M., Yang H., Pass H.I., Krausz T., Testa J.R., Gaudino G. (2013). BAP1 and cancer. Nat. Rev. Cancer.

[6] Bott M., Brevet M., Taylor B.S., Shimizu S., Ito T., Wang L., Creaney J., Lake R.A., Zakowski M.F., Reva B., Sander C., Delsite R., Powell S., Zhou Q., Shen R. (2011). The nuclear deubiquitinase BAP1 is commonly inactivated by somatic mutations and 3p21.1 losses in malignant pleural mesothelioma. Nat. Genet..

[7] Nasu M., Emi M., Pastorino S., Tanji M., Powers A., Luk H., Baumann F., Zhang Y.A., Gazdar A., Kanodia S., Tiirikainen M., Flores E., Gaudino G., Becich M.J., Pass H.I. (2015). High incidence of somatic BAP1 alterations in sporadic malignant mesothelioma. J. Thorac. Oncol..

[8] Lo Iacono M., Monica V., Righi L., Grosso F., Libener R., Vatrano S., Bironzo P., Novello S., Musmeci L., Volante M., Papotti M., Scagliotti G.V. (2015). Targeted next-generation sequencing of cancer genes in advanced stage malignant pleural mesothelioma: A retrospective study. J. Thorac. Oncol..

[9] Kato S., Tomson B.N., Buys T.P.H., Elkin S.K., Carter J.L., Kurzrock R. (2016). Genomic landscape of malignant mesotheliomas. Mol. Cancer Ther..

[10] Righi L., Duregon E., Vatrano S., Izzo S., Giorcelli J., Rondón-Lagos M., Ascoli V., Ruffini E., Ventura L., Volante M., Papotti M., Scagliotti G.V. (2016). BRCA1-associated protein 1 (BAP1) immunohistochemical expression as a diagnostic tool in malignant pleural mesothelioma classification: A large retrospective study. J. Thorac. Oncol..

[11] Ewens K.G., Lalonde E., Richards-Yutz J., Shields C.L., Ganguly A. (2018). Comparison of germline *versus* somatic BAP1 mutations for risk of metastasis in uveal melanoma. BMC Cancer.

[12] Robertson A.G., Shih J., Yau C., Gibb E.A., Oba J., Mungall K.L., Hess J.M., Uzunangelov V., Walter V., Danilova L., Lichtenberg T.M., Kucherlapati M., Kimes P.K., Tang M., Penson A. (2017). Integrative analysis identifies four molecular and clinical subsets in uveal melanoma. Cancer Cell.

[13] Smit K.N., Jager M.J., de Klein A., Kiliҫ E. (2020). Uveal melanoma: Towards a molecular understanding. Prog. Retin. Eye Res..

[14] Jiao Y., Pawlik T.M., Anders R.A., Selaru F.M., Streppel M.M., Lucas D.J., Niknafs N., Guthrie V.B., Maitra A., Argani P., Offerhaus G.J.A., Roa J.C., Roberts L.R., Gores G.J., Popescu I. (2013). Exome sequencing identifies frequent inactivating mutations in BAP1, ARID1A and PBRM1 in intrahepatic cholangiocarcinomas. Nat. Genet..

[15] Andrici J., Goeppert B., Sioson L., Clarkson A., Renner M., Stenzinger A., Tayao M., Watson N., Farzin M., Toon C.W., Smith R.C., Mittal A., Samra J.S., Hugh T.J., Chou A. (2016). Loss of BAP1 expression occurs frequently in intrahepatic cholangiocarcinoma. Medicine (Baltimore).

[16] Misumi K., Hayashi A., Shibahara J., Arita J., Sakamoto Y., Hasegawa K., Kokudo N., Fukayama M. (2017). Intrahepatic cholangiocarcinoma frequently shows loss of BAP1 and PBRM1 expression, and demonstrates specific clinicopathological and genetic characteristics with BAP1 loss. Histopathology.

[17] Dizman N., Philip E.J., Pal S.K. (2020). Genomic profiling in renal cell carcinoma. Nat. Rev. Nephrol..

[18] Joseph R.W., Kapur P., Serie D.J., Eckel-Passow J.E., Parasramka M., Ho T., Cheville J.C., Frenkel E., Rakheja D., Brugarolas J., Parker A. (2014). Loss of BAP1 protein expression is an independent marker of poor prognosis in patients with low-risk clear cell renal cell carcinoma. Cancer.

[19] Peña-Llopis S., Vega-Rubín-De-Celis S., Liao A., Leng N., Pavía-Jiménez A., Wang S., Yamasaki T., Zhrebker L., Sivanand S., Spence P., Kinch L., Hambuch T., Jain S., Lotan Y., Margulis V. (2012). BAP1 loss defines a new class of renal cell carcinoma. Nat. Genet..

[20] Lafave L.M., Béguelin W., Koche R., Teater M., Spitzer B., Chramiec A., Papalexi E., Keller M.D., Hricik T., Konstantinoff K., Micol J.B., Durham B., Knutson S.K., Campbell J.E., Blum G. (2015). Loss of BAP1 function leads to EZH2-dependent transformation. Nat. Med..

[21] Zauderer M.G., Szlosarek P.W., Le Moulec S., Popat S., Taylor P., Planchard D., Scherpereel A., Jahan T.M., Koczywas M., Forster M., Cameron R.B., Peikert T., Argon E.K., Michaud N., Yang J. (2020). Safety and efficacy of tazemetostat, an enhancer of zeste-homolog 2 inhibitor, in patients with relapsed or refractory malignant mesothelioma. J. Clin. Oncol..

[22] Bononi A., Giorgi C., Patergnani S., Larson D., Verbruggen K., Tanji M., Pellegrini L., Signorato V., Olivetto F., Pastorino S., Nasu M., Napolitano A., Gaudino G., Morris P., Sakamoto G. (2017). BAP1 regulates IP3R3-mediated Ca 2+ flux to mitochondria suppressing cell transformation. Nature.

[23] Kolluri K.K., Alifrangis C., Kumar N., Ishii Y., Price S., Michaut M., Williams S., Barthorpe S., Lightfoot H., Busacca S., Sharkey A., Yuan Z., Sage E.K., Vallath S., Le Quesne J. (2018). Loss of functional BAP1 augments sensitivity to TRAIL in cancer cells. Elife.

[24] Kischkel F.C., Lawrence D.A., Chuntharapai A., Schow P., Kim K.J., Ashkenazi A. (2000). Apo2L/TRAIL-dependent recruitment of endogenous FADD and caspase-8 to death receptors 4 and 5. Immunity.

[25] Abdulghani J., El-Deiry W.S. (2010). TRAIL receptor signaling and therapeutics. Expert Opin. Ther. Targets.

[26] Von Karstedt S., Montinaro A., Walczak H. (2017). Exploring the TRAILs less travelled: TRAIL in cancer biology and therapy. Nat. Rev. Cancer.

[27] Ashkenazi A., Pai R.C., Fong S., Leung S., Lawrence D.A., Marsters S.A., Blackie C., Chang L., McMurtrey A.E., Hebert A., DeForge L., Koumenis I.L., Lewis D., Harris L., Bussiere J. (1999). Safety and antitumor activity of recombinant soluble Apo2 ligand. J. Clin. Invest..

[28] Walczak H., Miller R.E., Ariail K., Gliniak B., Griffith T.S., Kubin M., Chin W., Jones J., Woodward A., Le T., Smith C., Smolak P., Goodwin R.G., Rauch C.T., Schuh J.A.C.L. (1999). Tumoricidal activity of tumor necrosis factor-related apoptosis-inducing ligand *in vivo*. Nat. Med..

[29] Prasad S., Kim J.H., Gupta S.C., Aggarwal B.B. (2014). Targeting death receptors for TRAIL by agents designed by Mother Nature. Trends Pharmacol. Sci..

[30] De Vries E.G.E., Gietema J.A., De Jong S. (2006). Tumor necrosis factor-related apoptosis-inducing ligand pathway and its therapeutic implications. Clin. Cancer Res..

[31] Van Roosmalen I.A.M., Quax W.J., Kruyt F.A.E. (2014). Two death-inducing human TRAIL receptors to target in cancer: Similar or distinct regulation and function?. Biochem. Pharmacol..

[32] Lemke J., Von Karstedt S., Zinngrebe J., Walczak H. (2014). Getting TRAIL back on track for cancer therapy. Cell Death Differ..

[33] Ashkenazi A. (2015). Targeting the extrinsic apoptotic pathway in cancer: Lessons learned and future directions. J. Clin. Invest..

[34] De Miguel D., Lemke J., Anel A., Walczak H., Martinez-Lostao L. (2016). Onto better TRAILs for cancer treatment. Cell Death Differ..

[35] Dianat-Moghadam H., Heidarifard M., Mahari A., Shahgolzari M., Keshavarz M., Nouri M., Amoozgar Z. (2020). TRAIL in oncology: From recombinant TRAIL to nano- and self-targeted TRAIL-based therapies. Pharmacol. Res..

[36] Zhang L., Fang B. (2005). Mechanisms of resistance to TRAIL-induced apoptosis in cancer. Cancer Gene Ther..

[37] Deng D., Shah K. (2020). TRAIL of hope meeting resistance in cancer. Trends Cancer.

[38] Kumar N., Alrifai D., Kolluri K.K., Sage E.K., Ishii Y., Guppy N., Borg E., Falzon M., Nankivell M., Nicholson A., Janes S.M. (2019). Retrospective response analysis of BAP1 expression to predict the clinical activity of systemic cytotoxic chemotherapy in mesothelioma. Lung Cancer.

[39] Rintoul R.C., Rassl D.M., Gittins J., Marciniak S.J. (2016). MesobanK UK: An international mesothelioma bioresource. Thorax.

[40] Chernova T., Sun X.M., Powley I.R., Galavotti S., Grosso S., Murphy F.A., Miles G.J., Cresswell L., Antonov A.V., Bennett J., Nakas A., Dinsdale D., Cain K., Bushell M., Willis A.E. (2016). Molecular profiling reveals primary mesothelioma cell lines recapitulate human disease. Cell Death Differ..

[41] Al-Taei S., Salimu J., Lester J.F., Linnane S., Goonewardena M., Harrop R., Mason M.D., Tabi Z. (2012). Overexpression and potential targeting of the oncofoetal antigen 5T4 in malignant pleural mesothelioma. Lung Cancer.

[42] Lincoln F.A., Imig D., Boccellato C., Juric V., Noonan J., Kontermann R.E., Allgöwer F., Murphy B.M., Rehm M. (2018). Sensitization of glioblastoma cells to TRAIL-induced apoptosis by IAP- and Bcl-2 antagonism. Cell Death Dis..

[43] Lee T.J., Lee J.T., Park J.W., Kwon T.K. (2006). Acquired TRAIL resistance in human breast cancer cells are caused by the sustained cFLIPL and XIAP protein levels and ERK activation. Biochem. Biophys. Res. Commun..

[44] Kaminskyy V.O., Surova O.V., Piskunova T., Zborovskaya I.B., Tchevkina E.M., Andera L., Zhivotovsky B. (2013). Upregulation of c-FLIP-short in response to TRAIL promotes survival of NSCLC cells, which could be suppressed by inhibition of Ca2+/calmodulin signaling. Cell Death Dis..

[45] Ishimura N., Isomoto H., Bronk S.F., Gores G.J. (2006). Trail induces cell migration and invasion in apoptosis-resistant cholangiocarcinoma cells. Am. J. Physiol. Gastrointest. Liver Physiol..

[46] Falschlehner C., Emmerich C.H., Gerlach B., Walczak H. (2007). TRAIL signalling: Decisions between life and death. Int. J. Biochem. Cell Biol..

[47] Screaton G.R., Mongkolsapaya J., Xu X.N., Cowper A.E., McMichael A.J., Bell J.I. (1997). TRICK2, a new alternatively spliced receptor that transduces the cytotoxic signal from TRAIL. Curr. Biol..

[48] Valley C.C., Lewis A.K., Mudaliar D.J., Perlmutter J.D., Braun A.R., Karim C.B., Thomas D.D., Brody J.R., Sachs J.N. (2012). Tumor necrosis factor-related apoptosis-inducing ligand (TRAIL) induces death receptor 5 networks that are highly organized. J. Biol. Chem..

[49] Tate J.G., Bamford S., Jubb H.C., Sondka Z., Beare D.M., Bindal N., Boutselakis H., Cole C.G., Creatore C., Dawson E., Fish P., Harsha B., Hathaway C., Jupe S.C., Kok C.Y. (2019). Cosmic: The catalogue of somatic mutations in cancer. Nucleic Acids Res..

[50] Bagci-Onder T., Agarwal A., Flusberg D., Wanningen S., Sorger P., Shah K. (2013). Real-time imaging of the dynamics of death receptors and therapeutics that overcome TRAIL resistance in tumors. Oncogene.

[51] Szegezdi E., Reis C.R., van der Sloot A.M., Natoni A., O’Reilly A., Reeve J., Cool R.H., O’Dwyer M., Knapper S., Serrano L., Quax W.J., Samali A. (2011). Targeting AML through DR4 with a novel variant of rhTRAIL. J. Cell. Mol. Med..

[52] Xiao W., Ishdorj G., Sun J., Johnston J.B., Gibson S.B. (2011). Death receptor 4 is preferentially recruited to lipid rafts in chronic lymphocytic leukemia cells contributing to tumor necrosis related apoptosis inducing ligand-induced synergistic apoptotic responses. Leuk. Lymphoma.

[53] Lemke J., Noack A., Adam D., Tchikov V., Bertsch U., Röder C., Schütze S., Wajant H., Kalthoff H., Trauzold A. (2010). TRAIL signaling is mediated by DR4 in pancreatic tumor cells despite the expression of functional DR5. J. Mol. Med..

[54] Van Geelen C.M.M., Pennarun B., Le P.T.K., De Vries E.G.E., De Jong S. (2011). Modulation of TRAIL resistance in colon carcinoma cells: Different contributions of DR4 and DR5. BMC Cancer.

[55] Baritaki S., Huerta-Yepez S., Sakai T., Spandidos D.A., Bonavida B. (2007). Chemotherapeutic drugs sensitize cancer cells to TRAIL-mediated apoptosis: Up-regulation of DR5 and inhibition of Yin Yang 1. Mol. Cancer Ther..

[56] Huerta-Yepez S., Vega M., Escoto-Chavez S.E., Murdock B., Sakai T., Baritaki S., Bonavida B. (2009). Nitric oxide sensitizes tumor cells to TRAIL-induced apoptosis *via* inhibition of the DR5 transcription repressor Yin Yang 1. Nitric Oxide.

[57] Yu H., Mashtalir N., Daou S., Hammond-Martel I., Ross J., Sui G., Hart G.W., Rauscher F.J., Drobetsky E., Milot E., Shi Y., Affar E.B. (2010). The ubiquitin carboxyl hydrolase BAP1 forms a ternary complex with YY1 and HCF-1 and is a critical regulator of gene expression. Mol. Cell. Biol..

[58] Greer Y.E., Gilbert S.F., Gril B., Narwal R., Peacock Brooks D.L., Tice D.A., Steeg P.S., Lipkowitz S. (2019). MEDI3039, a novel highly potent tumor necrosis factor (TNF)-related apoptosis-inducing ligand (TRAIL) receptor 2 agonist, causes regression of orthotopic tumors and inhibits outgrowth of metastatic triple-negative breast cancer. Breast Cancer Res..

[59] Okino Y., Machida Y., Frankland-Searby S., Machida Y.J. (2015). BRCA1-associated protein 1 (BAP1) deubiquitinase antagonizes the ubiquitin-mediated activation of FoxK2 target genes. J. Biol. Chem..

[60] Horak P., Pils D., Haller G., Pribill I., Roessler M., Tomek S., Horvat R., Zeillinger R., Zielinski C., Krainer M. (2005). Contribution of epigenetic silencing of tumor necrosis factor-related apoptosis inducing ligand receptor 1 (DR4) to TRAIL resistance and ovarian cancer. Mol. Cancer Res..

[61] Elias A., Siegelin M.D., Steinmüller A., Von Deimling A., Lass U., Korn B., Mueller W. (2009). Epigenetic silencing of death receptor 4 mediates tumor necrosis factor-related apoptosis-inducing ligand resistance in gliomas. Clin. Cancer Res..

[62] Jin Z., McDonald E.R., Dicker D.T., El-Deiry W.S. (2004). Deficient tumor necrosis factor-related apoptosis-inducing ligand (TRAIL) death receptor transport to the cell surface in human colon cancer cells selected for resistance to TRAIL-induced apoptosis. J. Biol. Chem..

[63] Martínez-Paniagua M.A., Baritaki S., Huerta-Yepez S., Ortiz-Navarrete V.F., González-Bonilla C., Bonavida B., Vega M.I. (2011). Mcl-1 and YY1 inhibition and induction of DR5 by the BH3-mimetic obatoclax (GX15-070) contribute in the sensitization of B-NHL cells to TRAIL apoptosis. Cell Cycle.

[64] Szczepanski A.P., Wang L. (2021). Emerging multifaceted roles of BAP1 complexes in biological processes. Cell Death Discov..

[65] Hoxha S., Shepard A., Troutman S., Diao H., Doherty J.R., Janiszewska M., Witwicki R.M., Pipkin M.E., Ja W.W., Kareta M.S., Kissil J.L. (2020). YAP-mediated recruitment of YY1 and EZH2 represses transcription of key cell-cycle regulators. Cancer Res..

[66] Garbán H.J., Bonavida B. (2001). Nitric oxide inhibits the transcription repressor Yin-Yang 1 binding activity at the silencer region of the Fas promoter: A pivotal role for nitric oxide in the up-regulation of Fas gene expression in human tumor cells. J. Immunol..

[67] Vega M.I., Jazirehi A.R., Huerta-Yepez S., Bonavida B. (2005). Rituximab-induced inhibition of YY1 and Bcl-x L expression in Ramos non-Hodgkin’s lymphoma cell line *via* inhibition of NF-κB activity: Role of YY1 and Bcl-x L in Fas resistance and chemoresistance, respectively. J. Immunol..

[68] Vega M.I., Huerta-Yepez S., Jazirehi A.R., Garban H., Bonavida B. (2005). Rituximab (chimeric anti-CD20) sensitizes B-NHL cell lines to Fas-induced apoptosis. Oncogene.

[69] Qin J., Zhou Z., Chen W., Wang C., Zhang H., Ge G., Shao M., You D., Fan Z., Xia H., Liu R., Chen C. (2015). BAP1 promotes breast cancer cell proliferation and metastasis by deubiquitinating KLF5. Nat. Commun..

[70] Jia X., Chen H., Ren Y., Dejizhuoga, Gesangyuzhen, Gao N., Feng H., Huang W., Liao Y., Yu H. (2021). BAP1 antagonizes WWP1-mediated transcription factor KLF5 ubiquitination and inhibits autophagy to promote melanoma progression. Exp. Cell Res..

[71] Liu X., Kumar M., Yang L., Molkentine D.P., Valdecanas D., Yu S., Meyn R.E., Heymach J.V., Skinner H.D. (2018). BAP1 is a novel target in HPV-negative head and neck cancer. Clin. Cancer Res..

[72] Guo Y., Yang H., Chen S., Zhang P., Li R., Nimer S.D., Harbour J.W., Xu M., Yang F.C. (2018). Reduced BAP1 activity prevents ASXL1 truncation-driven myeloid malignancy *in vivo*. Leukemia.

[73] Khachigian L.M. (2018). The Yin and Yang of YY1 in tumor growth and suppression. Int. J. Cancer.

[74] Meliala I.T.S., Hosea R., Kasim V., Wu S. (2020). The biological implications of Yin Yang 1 in the hallmarks of cancer. Theranostics.

[75] Mercer P.F., Woodcock H.V., Eley J.D., Platé M., Sulikowski M.G., Durrenberger P.F., Franklin L., Nanthakumar C.B., Man Y., Genovese F., McAnulty R.J., Yang S., Maher T.M., Nicholson A.G., Blanchard A.D. (2016). Exploration of a potent PI3 kinase/mTOR inhibitor as a novel anti-fibrotic agent in IPF. Thorax.

[76] Butler C.R., Hynds R.E., Gowers K.H.C., Lee D.D.H., Brown J.M., Crowley C., Teixeira V.H., Smith C.M., Urbani L., Hamilton N.J., Thakrar R.M., Booth H.L., Birchall M.A., De Coppi P., Giangreco A. (2016). Rapid expansion of human epithelial stem cells suitable for airway tissue engineering. Am. J. Respir. Crit. Care Med..

