# Supplementary material for: BAP1 and YY1 regulate expression of death receptors in malignant pleural mesothelioma
Source: J Biol Chem. 2021 Sep 29;297(5):101223. doi: 10.1016/j.jbc.2021.101223 (PMC8545693; doi:10.1016/j.jbc.2021.101223)
Supplement: Supplemental Figures S1–S12 [file mmc1.pdf]

Supporting Information

BAP1 and YY1 regulate expression of death receptors in malignant pleural mesothelioma

Yuki Ishii, Krishna K. Kolluri, Adam Pennycuick, Xidan Zhang, Ersilia Nigro, Doraid Alrifai, Elaine Borg, Mary Falzon, Khalid Shah, Neelam Kumar, Sam M. Janes

Figure S1: DR4 and DR5 expression is inversely correlated with nuclear BAP1 expression in MPM TMA

Figure S2: Expression levels of DR4 and DR5 are inversely correlated with BAP1 expression in patients with MPM tumours

Figure S3: Expression of proteins involved in TRAIL-induced signalling pathways in a panel of MPM cell lines

Figure S4: Loss of nuclear BAP1 expression correlates with increased DR4 and DR5 expression and increased rTRAIL sensitivity in early passage MPM cells

Figure S5: rTRAIL-resistance in BAP1-expressing cells is not due to the induction of anti-apoptotic proteins by rTRAIL treatment.

Figure S6: BAP1 knockdown increases TRAIL sensitivity in cancer cells

Figure S7: BAP1 knockdown does not increase death receptors expression and TRAIL sensitivity in normal cells

Figure S8: BAP1 negatively regulates expression of DR4 and DR5 leading to TRAIL resistance in H28 MPM cells

Figure S9: A list of transcription factors that potentially bind to DR4 and/or DR5 promoters

Figure S10: YY1 knockdown increases death receptors expression and TRAIL sensitivity in BAP1-wild-type-cancer cells

Figure S11: YY1 expression is not regulated by BAP1

Figure S12: Interaction of YY1 and HCF-1

Supporting Information Figure S1

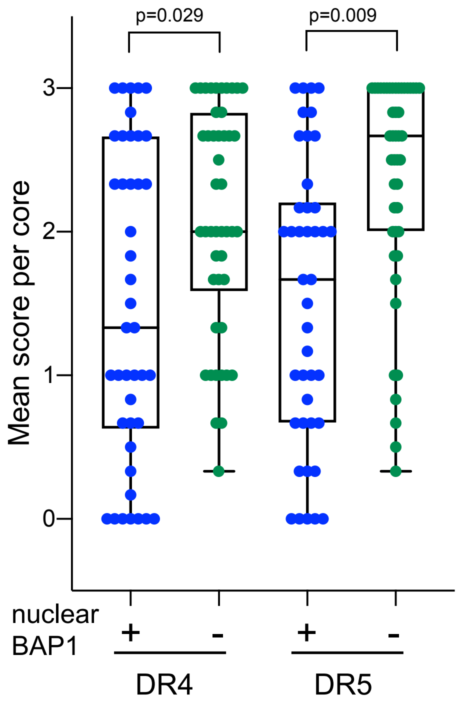

Figure S1: DR4 and DR5 expression is inversely correlated with nuclear BAP1 expression in MPM TMA

Semi-quantitative analysis of DR4 and DR5 expression in MPM TMA cores with (n=42) and without (n=46) nuclear BAP1 expression. Each dot represents an average score per core. P-values are calculated using a linear mixed models, accounting for the patient ID as a random effect. BAP1 positive samples expressed less DR4 (p=0.029) and DR5 (p=0.0091)

Supporting Information Figure S2

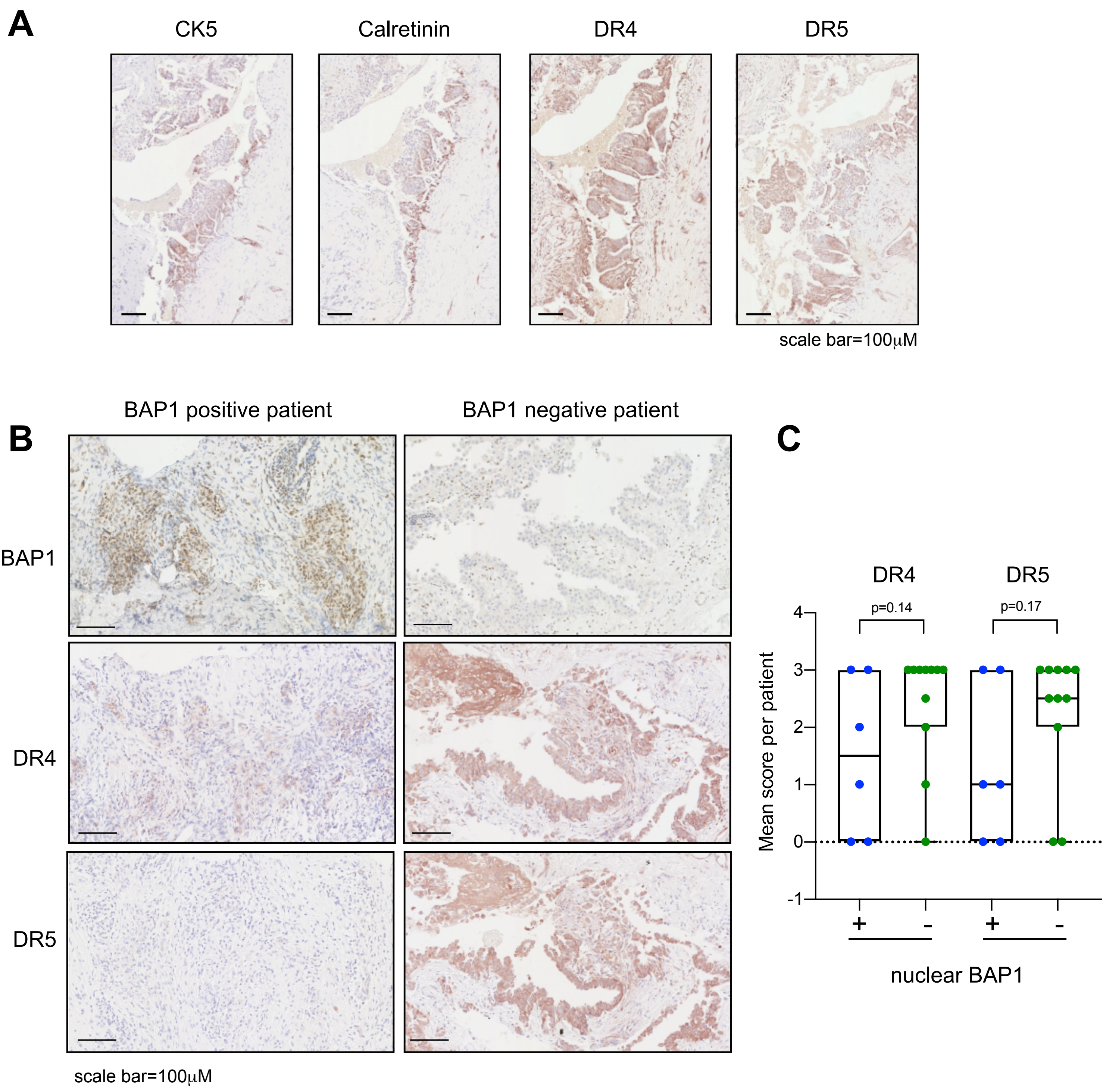

Figure S2: Expression levels of DR4 and DR5 are inversely correlated with BAP1 expression in patients with MPM tumours

A, Immunohistochemial analysis of DR4 and DR5 expression in primary MPM tissue. Two positive markers, cytokeratin 5 (CK5) and calretinin, were used to identify areas of MPM cells. Biopsy samples of 17 MPM patients (6 BAP1 positive, 11 BAP1-negative) from the MS01 clinical trial (NCT00075699) were used and representative images are shown. **B**, Immunohistochemical analysis of DR4, DR5 and BAP1 in primary MPM tissue as described in (A). Representative images show a strong inverse correlation between nuclear BAP1 expression and DR4/DR5 expression. **C**, Semi-quantitative analysis of IHC staining of DR4 and DR5 expression in primary MPM tissue with (+) (n=11) and without (-) (n=6) nuclear BAP1 staining. See method section for a detailed analysis.

Supporting Information Figure S3

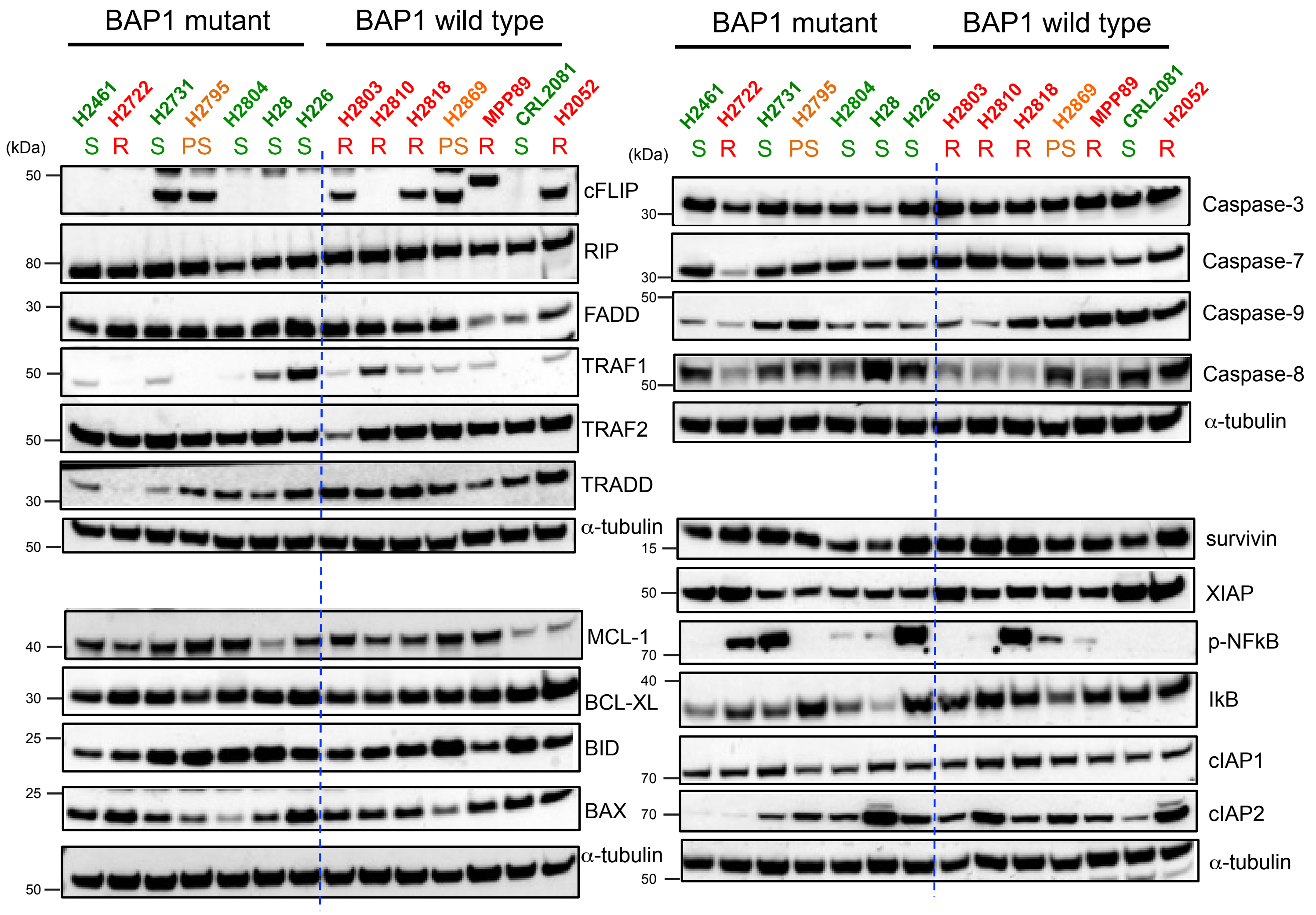

Figure S3: Expression of proteins involved in TRAIL-induced signalling pathways in a panel of MPM cell lines

MPM cell lines were divided into two groups (BAP1 mutant and BAP1 wild type) and are coloured according to the sensitivity to rTRAIL (green=sensitive (S); orange=partially sensitive (PS); red=resistant (R)). Proteins involved in TRAIL-induced signalling pathways, including pro- and anti-apoptotic pathways, were determined by immunoblots.

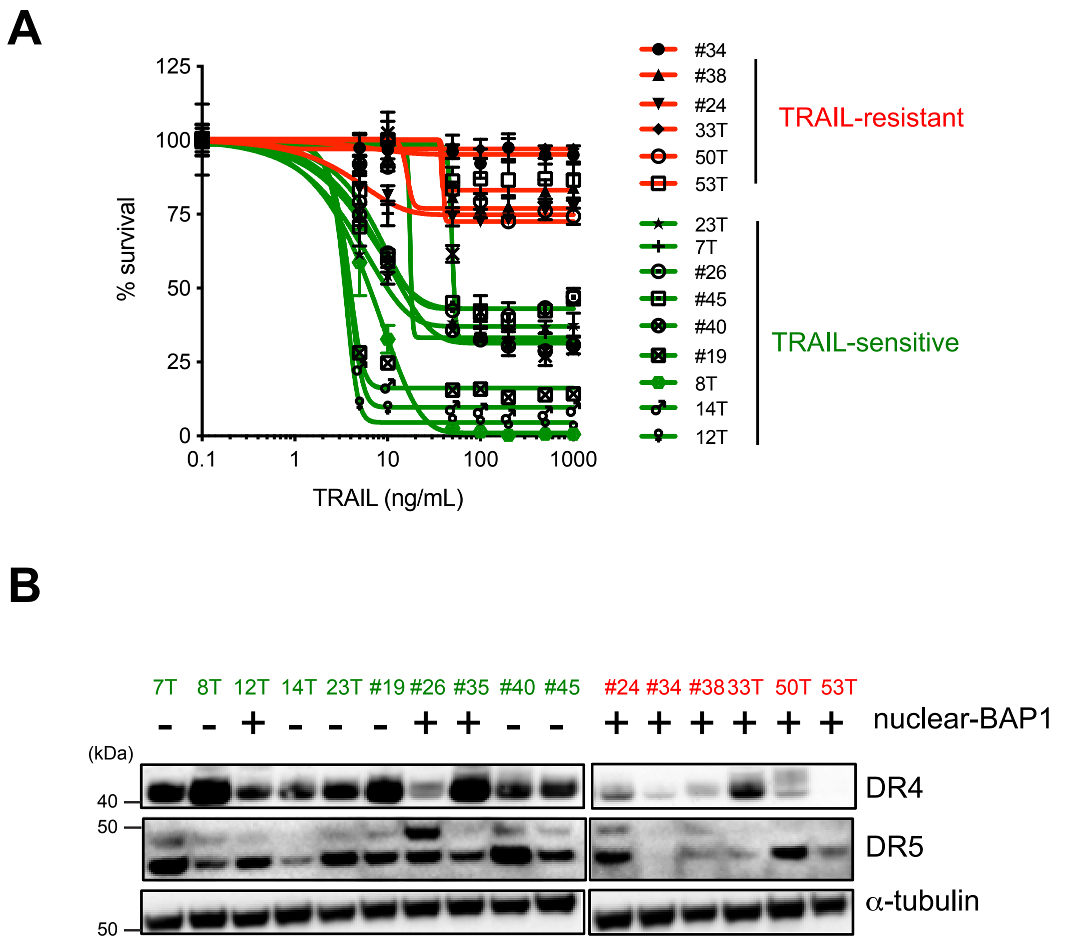

Figure S4: Loss of nuclear BAP1 expression correlates with increased DR4 and DR5 expression and increased rTRAIL sensitivity in early passage MPM cells

A, Cell viability assay of early passage MPM cells following treatment with a dose range of rTRAIL (0-1000ng/ml) for 72 hours. Cells with IC50>100ng/mL are defined as TRAIL-resistant (red); cells with IC50<100ng/mL are defined as TRAIL-sensitive (green). **B**, Immunoblots of DR4 and DR5 expression in early passage MPM cells stratified by rTRAIL sensitivity and the presence (+) or absence (-) of nuclear BAP1 expression Quantitative analysis of this experiment can be found in Fig.1E.

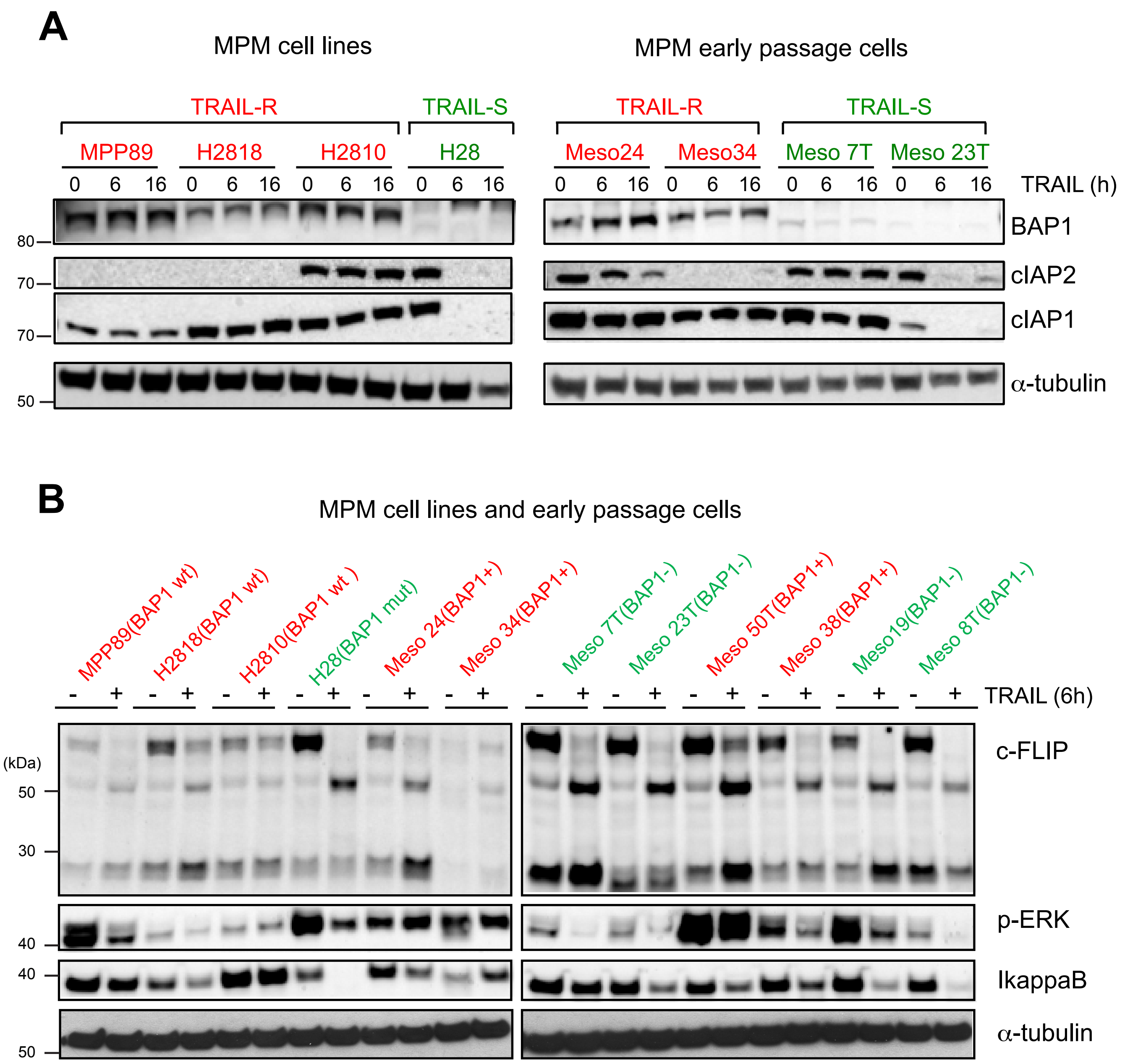

Figure S5: rTRAIL-resistance in BAP1-expressing cells is not due to the induction of anti-apoptotic proteins by rTRAIL treatment.

MPM cell lines and human early passage MPM cells were treated with 100ng/ml of rTRAIL for 0, 6 or 16 hours (**A**) or 0, 6 hours (**B**) to determine the expression of inhibitors of apoptosis proteins (cIAP1 and cIAP2), c-FLIP, p-ERK and IkappaB by western blot. Sensitivity to rTRAIL treatment is indicated as font colour: green sensitive (S); red resistant (R). BAP1's mutational status (wild type; wt or mutant; mut) or nuclear BAP1 expression (BAP1+ or BAP1-), which reflects the mutational status, is indicated (**B**).

Supporting Information Figure S6

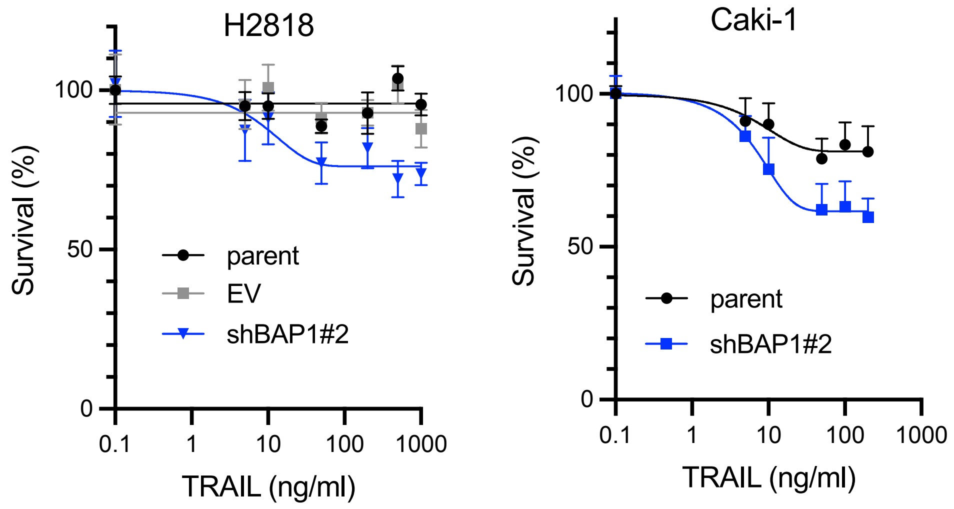

Figure S6: BAP1 knockdown increases TRAIL sensitivity in cancer cells

Cell viability assay in BAP1-wild-type MPM cells (H2818) and BAP1-wild-type- clear cell renal cell carcinoma (CCRCC) cells (Caki-1) transduced with BAP1 (shBAP1-clone#2) or empty vector (EV) shRNA. The cells were treated with a dose range of rTRAIL (0-1000ng/ml) for 72 hours, relative viability was assesed by MTT assay.

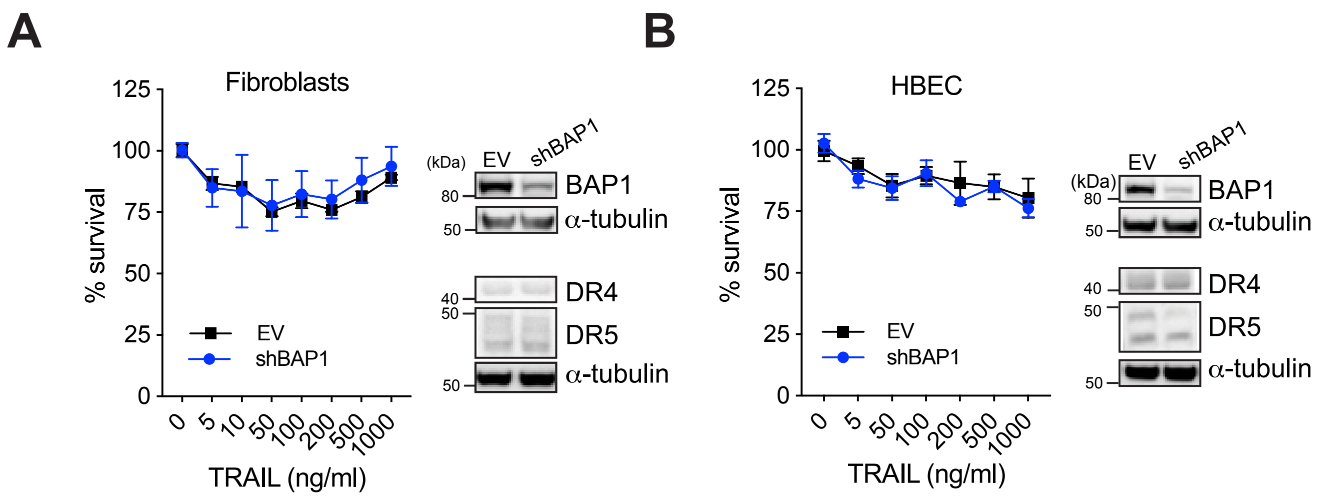

Figure S7: BAP1 knockdown does not increase death receptors expression and TRAIL sensitivity in normal cells

A& B, Cell viability assay of fibroblasts (**A**) and human bronchial epithelial cells (HBECs) (**B**) transduced with EV or shBAP1 (clone#1) following treatment with a dose range of rTRAIL (0-1000ng/ml) for 72 hours. Immunoblots of BAP1, DR4 and DR5 in these cells are also shown. Error bars represent the standard deviation.

Supporting Information Figure S8

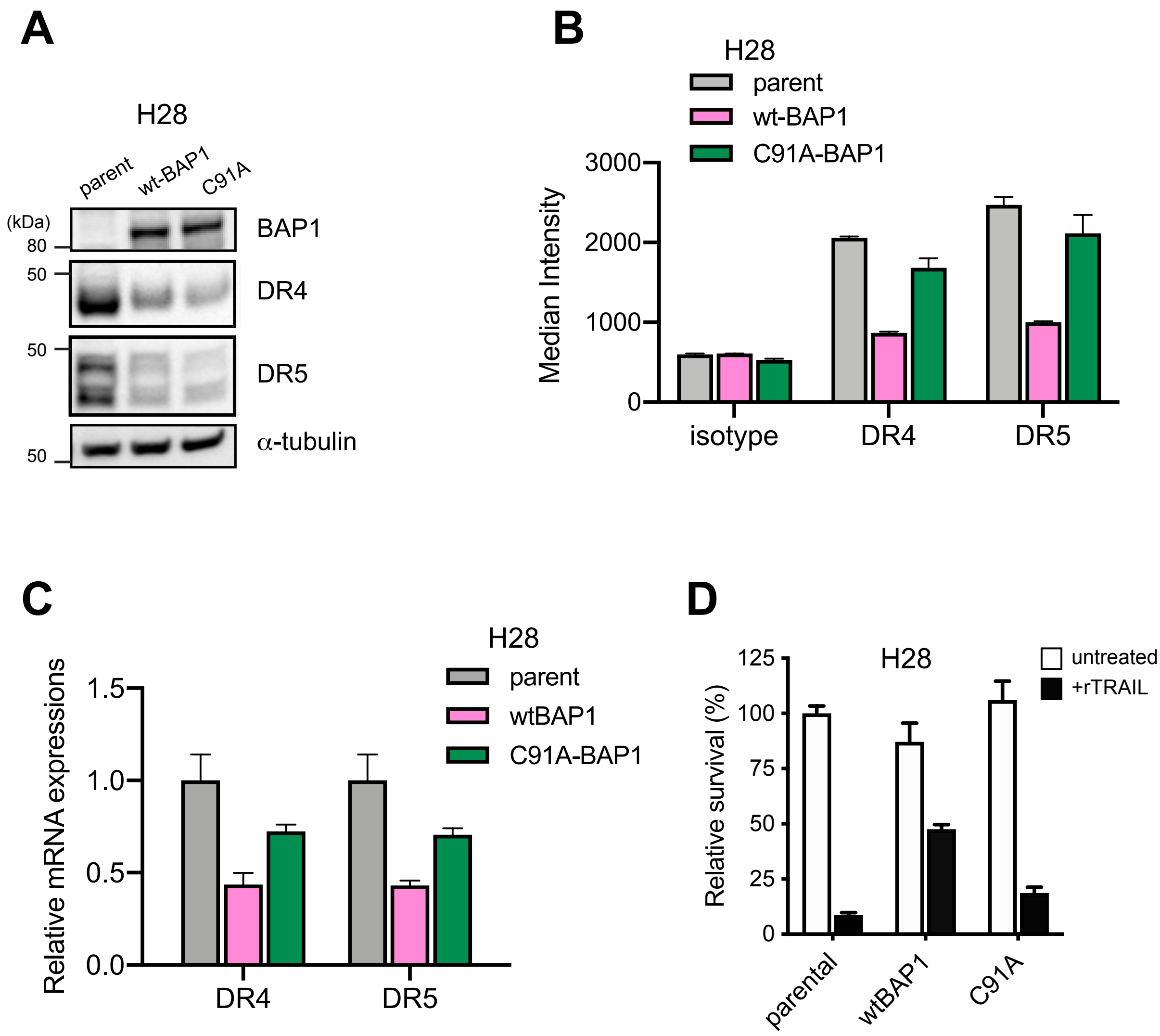

Figure S8: BAP1 negatively regulates expression of DR4 and DR5 leading to TRAIL resistance in H28 MPM cells

A, Immunoblot of BAP1- null H28 cells transduced with wild-type-BAP1 (wt-BAP1) or catalytically inactive BAP1-mutant (C91A-BAP1). **B**, Flow cytometry analysis of DR4 and DR5 cell surface expression in H28 cells transduced with wt-BAP1 or C91A-BAP1. Data shown are the mean ± s.d. performed in triplicates. **C**, Quantitative PCR analysis of DR4 and DR5 mRNA in H28 cells transduced with wt-BAP1 or C91A-BAP1. Data shown are the mean ± s.d. performed in triplicates. **D**, The sensitivity to rTRAIL (50ng/mL) is decreased in wt-BAP1-transduced H28 cells. Cell viability was assessed 72 hours later by XTT assay. Data shown are the mean ± s.d. (n=6).

only DR4	only DR5	Both DR4&DR5
NR3C1	USF2	YY1
HNF1A	NF1	NFKB1
IRF2	ETS2.1	TP53
USF2	NFKB1.1	FOXA1
RARA		CEBPB
ELF1		NFIC
RXRA.2		TAF6
ARNT		XBP1
		EBF2
		RXRA
		RXRA.1
		NFYA
		E2F1
		ATF3
		ETS2
		ETFB
		STAT1
		POU2F1
		SP1
		JUN
		GATA2
		LEF1
		RELA
		ELK1

Figure S9: A list of transcription factors that potentially bind to DR4 and/or DR5 promoters

The promoter sequences, that are 2000 bases upstream of the coding region, were put into an online tool, Human Core-Promoter Finder (http://rulai.cshl.org/tools/genefinder/CPROMOTER/human.htm), to find transcription factors predicted to bind DR4 and/or DR5.

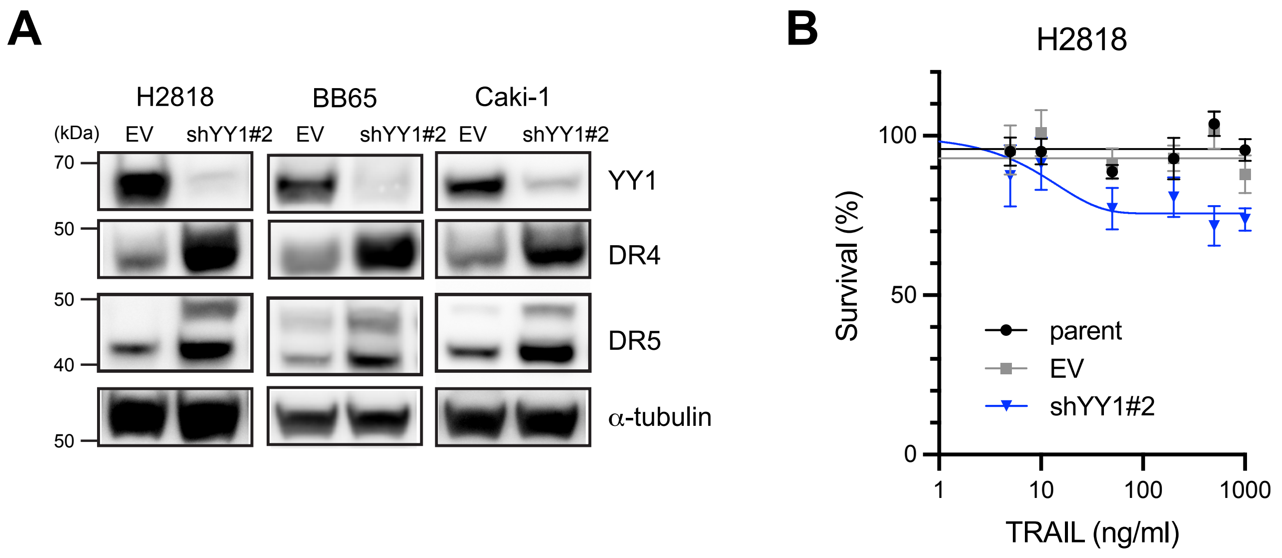

Figure S10: YY1 knockdown increases death receptors expression and TRAIL sensitivity in BAP1-wild-type-cancer cells

A, Immunoblots of YY1, DR4 and DR5 in BAP1-wild-type MPM cells (H2818) and in BAP1-wild-type- clear cell renal cell carcinoma (CCRCC) cells (BB65 and Caki-1) transduced with YY1 (shYY1-clone#2) or empty vector (EV) shRNA. **B**, Cell viability assay of parental, EV- or shYY1(clone#2) - transduced H2818 cells following treatment with a dose range of rTRAIL (0-1000ng/ml) for 72 hours.

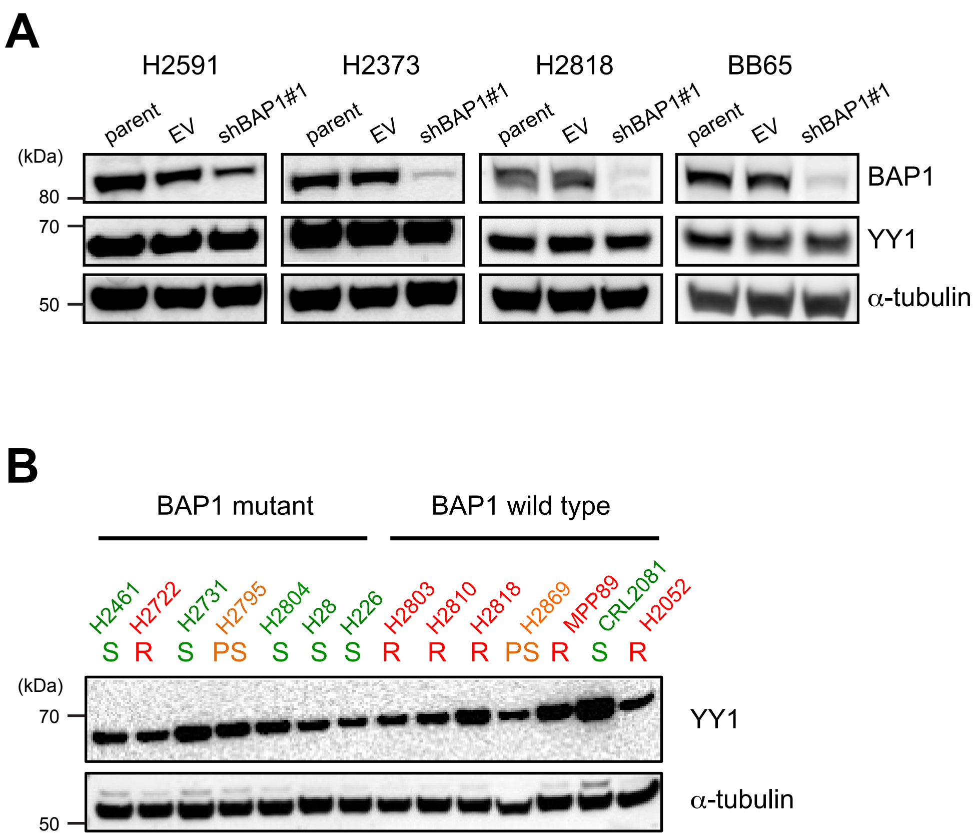

Figure S11: YY1 expression is not regulated by BAP1

A, Immunoblot analysis in parental, BAP1 shRNA (shBAP1 clone#1) or empty vector shRNA (EV) -transduced H2591, H2373, H2818 and BB65 cells. **B**, Immunoblot of YY1 expression in BAP1 mutant (n=7) vs BAP1 wild-type (n=7) MPM cells. Sensitivity to rTRAIL treatment is indicated as font colour: green sensitive (S); orange partially sensitive (PS); red resistant (R).

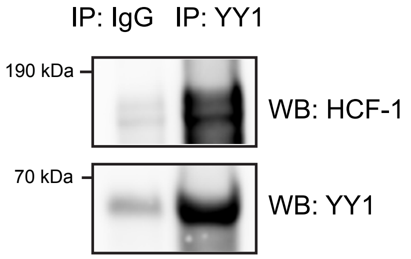

Figure S12: Interaction of YY1 and HCF-1

Co-Immunoprecipitation (Co-IP) of endogenous YY1 and HCF-1 in BAP1-wild type CCRCC (Caki-1) cells. Normal rabbit control IgG was used to show the specific interaction with the antibody.
